# Gut microbiome-related effects of berberine and probiotics on type 2 diabetes (the PREMOTE study)

**DOI:** 10.1038/s41467-020-18414-8

**Published:** 2020-10-06

**Authors:** Yifei Zhang, Yanyun Gu, Huahui Ren, Shujie Wang, Huanzi Zhong, Xinjie Zhao, Jing Ma, Xuejiang Gu, Yaoming Xue, Shan Huang, Jialin Yang, Li Chen, Gang Chen, Shen Qu, Jun Liang, Li Qin, Qin Huang, Yongde Peng, Qi Li, Xiaolin Wang, Ping Kong, Guixue Hou, Mengyu Gao, Zhun Shi, Xuelin Li, Yixuan Qiu, Yuanqiang Zou, Huanming Yang, Jian Wang, Guowang Xu, Shenghan Lai, Junhua Li, Guang Ning, Weiqing Wang

**Affiliations:** 1 National Clinical Research Centre for Metabolic Diseases, Ruijin Hospital, Shanghai Jiao Tong University School of Medicine, Shanghai, 200025 China; 2 BGI-Shenzhen, Shenzhen, 518083 China; 3 Dalian Institute of Chemical Physics, Chinese Academy of Science, Dalian, Liaoning Province China; 4 Ren Ji Hospital, Shanghai Jiao Tong University School of Medicine, Shanghai, China; 5 The First Affiliated hospital of Wenzhou Medical University, Zhejiang Province, China; 6 Nanfang Hospital, Southern Medical University, Guangdong Province, China; 7 Tong Ren Hospital, Shanghai Jiao Tong University School of Medicine, Shanghai, China; 8 Central Hospital of Minhang District, Shanghai, China; 9 Qilu Hospital of Shandong University, Shandong Province, China; 10 Fujian Provincial Hospital, Fujian Province, China; 11 Shanghai Tenth People’s Hospital of Tong Ji University, Shanghai, China; 12 Xuzhou Central Hospital, Jiangsu Province, China; 13 Xin Hua Hospital, Shanghai Jiao Tong University School of Medicine, Shanghai, China; 14 Chang Hai Hospital, Second Military Medical University, Shanghai, China; 15 Shanghai First People’s Hospital, Shanghai Jiao Tong University School of Medicine, Shanghai, China; 16 James D. Watson Institute of Genome Sciences, Hangzhou, Zhejiang Province China; 17 Johns Hopkins University School of Medicine, Baltimore, Maryland USA; 18 School of Biology and Biological Engineering, South China University of Technology, Guangzhou, Guangdong Province China

**Keywords:** Metagenomics, Metagenomics, Type 2 diabetes, Type 2 diabetes

## Abstract

Human gut microbiome is a promising target for managing type 2 diabetes (T2D). Measures altering gut microbiota like oral intake of probiotics or berberine (BBR), a bacteriostatic agent, merit metabolic homoeostasis. We hence conducted a randomized, double-blind, placebo-controlled trial with newly diagnosed T2D patients from 20 centres in China. Four-hundred-nine eligible participants were enroled, randomly assigned (1:1:1:1) and completed a 12-week treatment of either BBR-alone, probiotics+BBR, probiotics-alone, or placebo, after a one-week run-in of gentamycin pretreatment. The changes in glycated haemoglobin, as the primary outcome, in the probiotics+BBR (least-squares mean [95% CI], −1.04[−1.19, −0.89]%) and BBR-alone group (−0.99[−1.16, −0.83]%) were significantly greater than that in the placebo and probiotics-alone groups (−0.59[−0.75, −0.44]%, −0.53[−0.68, −0.37]%, P < 0.001). BBR treatment induced more gastrointestinal side effects. Further metagenomics and metabolomic studies found that the hypoglycaemic effect of BBR is mediated by the inhibition of DCA biotransformation by *Ruminococcus bromii*. Therefore, our study reports a human microbial related mechanism underlying the antidiabetic effect of BBR on T2D. (Clinicaltrial.gov Identifier: NCT02861261).

## Abstract (web summary)

The gut microbiome affects systemic metabolism and is a therapeutic target for type 2 diabetes. Here the authors demonstrate in a randomized controlled trial that effects of berberine, a plant alkaloid known to lower blood glucose, may be explained by the inhibition of *Ruminococcus bromii* mediated biotransformation of the bile acid deoxycholic acid.

## Introduction

The complex pathophysiology of type 2 diabetes (T2D) has posed a major challenge to the control of hyperglycaemia and diabetes-related mortality and morbidity^1–3^. In the past decade, the key role of gut microbiota in regulating host metabolism and the associations of gut microbial dysbiosis with the development of obesity and diabetes has been extensively explored^4–10^. Evidence from both human and animal studies has suggested that the gut microbiome serves as the common route to mediate the therapeutic effects of bariatric surgery, diet control and antidiabetic medications^4,11–15^. Several bacterial metabolic pathways regulating the production or transport of amino acids (aromatic, branched-chain amino acids and intermediates of histidine degradation)^16,17^, short-chain fatty acids (SCFAs)^18–20^ and bile acids (BAs)^16,21,22^ have been implicated in mediating bacterial regulation of host metabolic homoeostasis. Recent evidence has shown that both of the oral antidiabetic medications, metformin^15^ and acarbose^13^, can inhibit microbial BA metabolism by altering gut microbiome symbiosis and block gut BA signalling, thereby partially exerting their metabolic benefits. Interestingly, BA signalling has been proven to be required for gut microbiome-induced obesity and mediates the therapeutic effect of bariatric surgery^22–24^. Thus, the gut microbiome and microbial BA signalling, in particular, have become elusive targets for treating T2D.

The hunt for the microbial targeted remedies for T2D or other metabolic diseases has gained increasing attention. In records of Ayurvedic medicine in India and traditional medicine in China, berberine (BBR), a natural plant alkaloid extracted from *Berberis aristata* and *Coptis chinensis* (Huanglian), as an ancient antidiarrhoeal medication, has been reported to be an effective remedy for metabolic disorders, including T2D, by promoting liver lipid metabolism or adipose browning^25–27^. However, similar to metformin, the specific in vivo target of BBR has barely been clarified and its poor oral bioavailability has suggested a potential effect on the gut microbiome. 16S rRNA gene-sequencing studies in rodents have shown significant gut microbiota alterations induced by BBR and several microbial-related mechanisms, including the potential to alter SCFA and BA metabolism, have been found to underlie the metabolic benefits of BBR^28–31^. However, how the human gut microbiome responds to BBR treatment and how the microbial alterations are related to the metabolic benefits of BBR have not yet been investigated.

The potential for using probiotics to treat metabolic or other diseases constitutes another heated topic in gut microbiome studies. The inconsistent usage of strains and formulas, the heterogeneity of the target population and various qualities and validities across the studies might be the reasons for the controversial results of probiotic intervention^32–34^. Interestingly, studies, including ours, have revealed that indigenous probiotics containing genera such as *Lactobacillus* and *Bifidobacterium* are enriched in faeces from T2D participants after antidiabetic treatment with a single use of acarbose^14^ or metformin^11,12^, which are associated with an antidiabetic effect, but reported to be inhibited by BBR administration^31^. Hence, it prompts a possibility whether the application of probiotics together with a treatment such as BBR could confer superior antidiabetic benefits than using probiotics or BBR alone.

Therefore, aiming to find an effective strategy for treating T2D by altering gut microbiome dysbiosis, we have designed and conducted the Probiotics and BBR on the Efficacy and Change of Gut Microbiota in Patients with Newly Diagnosed Type 2 Diabetes (PREMOTE) trial. The primary objective of the trial is to determine and compare the efficacy of probiotics + BBR (Prob + BBR), BBR + placebo (BBR) or probiotics + placebo (Prob), to that of placebo (Plac) in reducing glycaemic haemoglobin (HbA1c) among participants diagnosed with T2D. The secondary outcomes, including clinical metabolic measurements, are also evaluated and compared across the groups. Comprehensive metagenomics and metabolomics analyses are employed to investigate the potential for regulating the gut microbiome of BBR and/or probiotics treatments, and how these gut microbial changes correlated with the antidiabetic effect after a 7-day antibiotic pretreatment.

## Results

### Participants and clinical outcomes after intervention

**Fig. 1:**
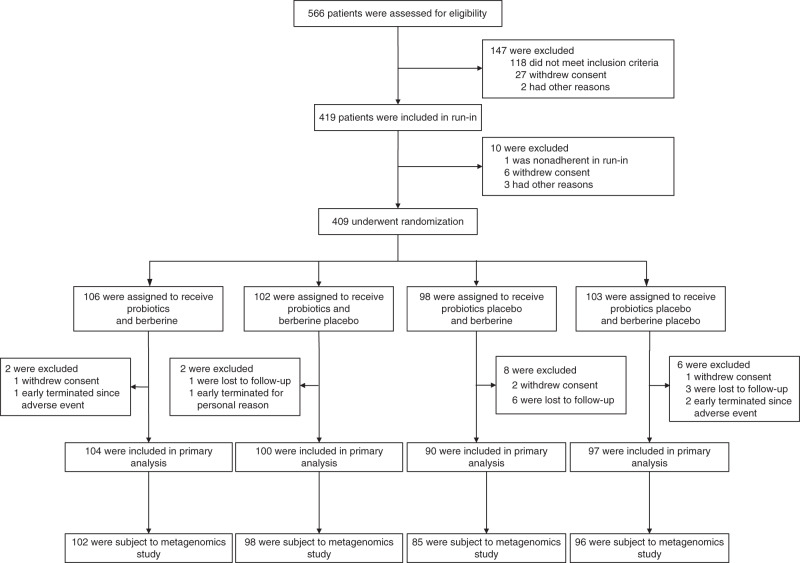
Flow diagram of participant enrolment in the PREMOTE Trial.

A total of 566 participants were screened for eligibility from 18 August 2016 to 18 July 2017, of whom 409 eligible participants were randomized with 106 in the Prob + BBR group, 102 in the Prob group, 98 in the BBR group and 103 in the Plac group (Fig. 1). The baseline characteristics of the participants were similar among the four groups (Table 1). By the end of the intervention, a total of 391 participants were included in the primary analysis. For the primary outcome, the change in HbA1c showed a significant difference between the four treatment groups (*P* < 0.001). The reduction in HbA1c at week 13 in the Prob + BBR group (least-squares mean [95% confidence interval, 95% CI], −1.04 [−1.19, −0.89]%) or BBR group (−0.99 [−1.16, −0.83]%) was significantly greater than that in the Plac group (−0.59 [−0.75, −0.44]%, both *P* < 0.001) and the Prob group (−0.53 [−0.68, −0.37]%, both *P* < 0.001), but no difference was found between those of the Prob + BBR and BBR groups (*P* = 0.70) or between the Prob and Plac groups (*P* = 0.53) (Table 2). Generalized estimating equation (GEE) analysis adjusted for confounding factors according to the protocol yielded similar results (Supplementary Table 1). Thus, BBR and BBR with probiotics were both superior to the Plac in lowering HbA1c, but Prob was not.

**Table 1:** Demographics and baseline characteristics of the randomized participants in Intervention Groups.

Characteristic	Plac (*n* = 103)	Prob (*n* = 102)	BBR (*n* = 98)	Prob + BBR (*n* = 106)
Age (IQR), years	54 (46–61)	54 (45–59)	53 (42–61)	53.5 (47–60)
Male sex, no. (%)	61 (59.2)	65 (63.7)	59 (60.2)	60 (56.6)
Diabetes duration, months	5 (3–9)	5 (3–10)	6 (3–11)	5 (3–11)
Body weight, kg	72.1 ± 12.5	71.9 ± 11.8	71.1 ± 13.6	70.9 ± 11.1
Body mass index, kg m^−2a^	26.2 ± 3.43	25.6 ± 2.96	25.7 ± 3.43	25.5 ± 2.86
Waist circumference, cm	91.9 ± 9.0	91.6 ± 8.5	90.7 ± 9.6	90.6 ± 8.2
Systolic blood pressure, mm Hg	129.0 ± 14.2	128.6 ± 13.8	127.9 ± 14.5	126.0 ± 12.7
Diastolic blood pressure, mm Hg	80.3 ± 8.9	80.5 ± 8.3	79.3 ± 9.3	78.7 ± 9.0
HbA1c, %^b^	7.81 ± 0.81	7.78 ± 0.82	7.68 ± 0.76	7.66 ± 0.82
HbA1c, mmol/mol^c^	61.86 ± 14.64	61.53 ± 14.54	60.44 ± 15.19	60.22 ± 14.54
Fasting plasma glucose, mmol L^−1^	8.13 ± 1.48	8.39 ± 1.55	8.16 ± 1.55	8.07 ± 1.32
Post-load plasma glucose, mmol L^−1^	15.04 ± 2.58	14.80 ± 3.29	14.22 ± 3.23	14.39 ± 3.17
Fasting serum insulin (IQR), μIU ml^−1^	11.75 (8.00–17.43)	10.67 (8.55–15.16)	9.96 (7.00–16.03)	10.10 (7.54–14.00)
Post-load serum insulin (IQR), μIU ml^−1^	48.12 (35.99–82.90)	45.27 (34.33–64.60)	54.63 (31.27–69.77)	44.40 (32.35–64.33)
Fasting serum C peptide (IQR), ng ml^−1^	2.73 (2.08–3.38)	2.50 (2.08–3.29)	2.53 (1.99–3.08)	2.43 (2.11–3.25)
Post-load serum C peptide (IQR), ng ml^−1^	7.65 (5.86–9.58)	7.06 (5.69–8.79)	7.52 (5.83–9.16)	7.11 (5.75–9.59)
Triglyceride (IQR), mmol L^−1^	1.44 (1.06–1.91)	1.51 (1.02–2.40)	1.54 (1.09–2.26)	1.66 (1.19–2.35)
Total cholesterol, mmol L^−1^	5.18 ± 0.97	5.24 ± 1.04	4.99 ± 1.06	5.25 ± 0.94
HDL cholesterol, mmol L^−1^	1.25 ± 0.28	1.20 ± 0.27	1.22 ± 0.28	1.19 ± 0.23
LDL cholesterol, mmol L^−1^	3.33 ± 0.84	3.42 ± 0.86	3.22 ± 0.89	3.39 ± 0.80
HOMA-IR (IQR)^d^	4.45 (3.05–5.77)	4.14 (2.98–5.70)	3.74 (2.60–5.67)	3.51 (2.55–4.85)
HOMA-β (IQR)^e^	53.46 (33.10–91.25)	44.07 (33.55–70.10)	52.09 (31.44–76.21)	49.62 (29.76–75.24)

*BBR* berberine, *IQR* interquartile range, *Prob* Probiotics. No significant differences were observed among the four groups in any of the baseline characteristics. Data were presented as mean ± SD or median (IQR).

^a^Body mass index (BMI) is the weight in kilograms divided by the square of the height in metres.

^b^HbA1c is glycated haemoglobin, shown as the DCCT (Diabetes Control and Complications Trial) units.

^c^HbA1c is glycated haemoglobin, shown as the IFCC (International Federation of Clinical Chemistry) units.

^d^HOMA-IR refers to (Fasting serum insulin (μIU ml^−1^) × Fasting plasma glucose (mmol L^−^^1^))/22.5, homoeostasis model assessment index for assessing insulin resistance.

^e^HOMA-β, refer to (20 × Fasting serum insulin (μIU ml^−1^))/(Fasting plasma glucose (mmol L^−1^)− 3.5), homoeostasis model assessment index for assessing β-cell function.

**Table 2:** Primary outcomes in all and older participants (age ≥ 50 years).

		HbA1c 0 w (%)	HbA1c 13 w (%)	Change in HbA1c (95% CI)^a^	Change in HbA1c (95% CI)^b^	Model 1		Model 2	
						*P*-value^c^	*P*-value^d^	*P*-value^c^	*P*-value^d^
All participants									
Plac		7.81 ± 0.81	7.23 ± 0.97	−0.59 (−0.75, −0.44)		/	5.99E − 04	/	6.31E − 04
Prob		7.78 ± 0.82	7.27 ± 0.90	−0.53 (−0.68, −0.37)	0.07 (−0.20, 0.34)	0.53	5.15E − 05	0.52	5.11E − 05
BBR		7.68 ± 0.76	6.71 ± 0.77	−0.99 (−1.16, −0.83)	−0.40 (−0.67, −0.13)	5.99E − 04	/	6.31E − 04	/
Prob + BBR		7.66 ± 0.82	6.62 ± 0.66	−1.04 (−1.19, −0.89)	−0.44 (−0.71, −0.18)	8.41E − 05	0.70	6.99E − 05	0.66
Age ≥ 50 years									
Plac		7.76 ± 0.77	7.24 ± 1.13	−0.59 (−0.78, −0.39)		/	0.03	/	/
Prob		7.64 ± 0.69	7.07 ± 0.61	−0.52 (−0.72, −0.33)	0.06 (−0.26, 0.39)	0.65	8.65 E − 03	/	/
BBR		7.58 ± 0.69	6.82 ± 0.81	−0.90 (−1.10, −0.70)	−0.31 (−0.65, 0.02)	0.03	/	/	/
Prob + BBR		7.61 ± 0.73	6.62±0.57	−0.99 (−1.17, −0.82)	−0.41 (−0.72, −0.09)	2.39E − 03	0.48	/	/

*BBR* berberine treatment, *HbA1c* glycated haemoglobin, *Plac* placebo, *Prob* probiotics treatment, *Prob* *+* *BBR* berberine plus probiotics treatment.

Data were presented as mean ± SD. Model 1: analysis of variance (ANOVA) were performed to compare the Change in HbA1c between groups. Model 2: Multivariate ANOVA were performed to compare the Change in HbA1c between groups adjust for age group (age group defined as <50 and ≥50 years).

All *P*-values reported were two-sided for multiple comparisons using Bonferroni correction. A statistical significance level was set at *P* < 0.008.

^a^The values are least-squares means.

^b^Placebo subtracted change in HbA1c, least-squares means.

^c^*P*-values refer to comparison of change in HbA1c between Plac group and the other groups using ANOVA on the basis of intention-to-treat (ITT) analysis.

^d^*P*-values refer to comparison of change in HbA1c between BBR group and the other groups using ANOVA on the basis of ITT analysis.

Similar improvements were found in the other metabolic parameters (secondary outcomes) by BBR containing treatments, such as fasting plasma glucose (FPG), post-load plasma glucose (PPG), blood triglycerides (TGs), total cholesterol (TC) and low-density lipoprotein (LDL) cholesterol levels, except for homoeostasis model assessment index for insulin resistance (HOMA-IR), which was significantly lowered by Prob + BBR but not by BBR (Supplementary Table 2). More cases of gastrointestinal adverse effect (AE) cases occurred in both BBR arms and glycaemic control did not differ in participants with gastrointestinal AEs. All other AEs were comparable between the intervention and Plac groups (Supplementary Table 3) with normal hepatic and renal function after treatment (Supplementary Table 4). Subgroup analyses showed that diabetes duration and gastrointestinal AEs did not affect the primary outcome in our study (Supplementary Tables 5 and 6, post-hoc analysis).

### Metagenomic analysis showed a significant impact of BBR on the human gut microbiome

**Fig. 2:**
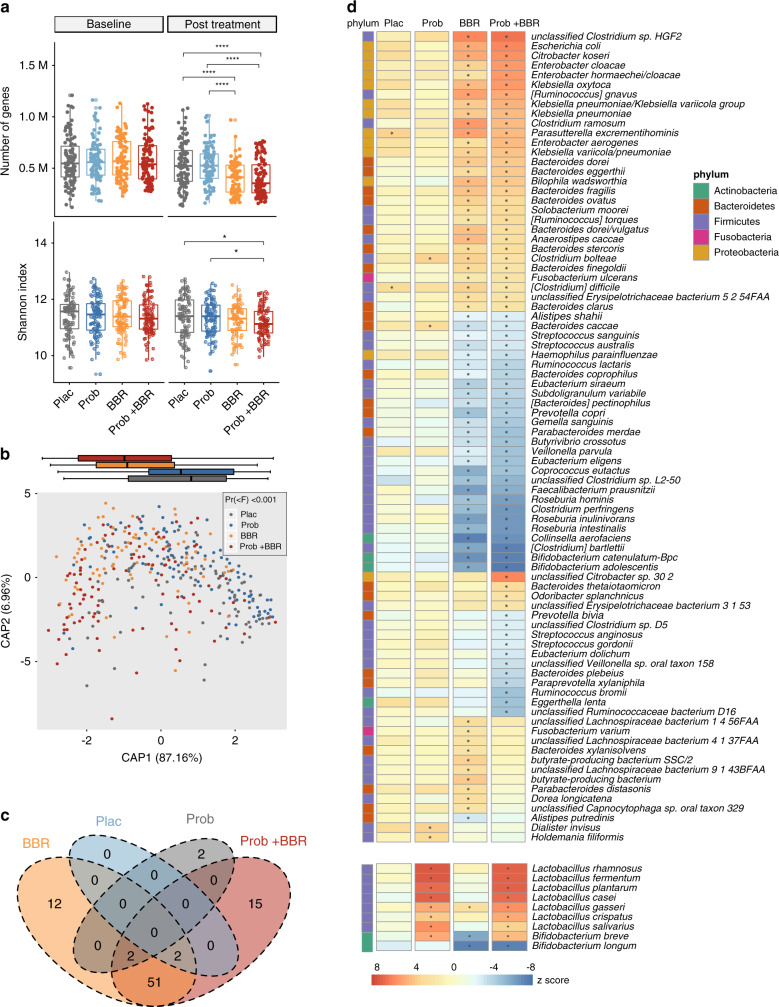
BBR significantly altered gut microbiome symbiosis after 13 weeks of treatment. **a** Gene count (upper panel) and Shannon index (lower panel) of genes in different arms, baseline and post treatment; Plac, Placebo, *n* = 96; Prob, probiotics treatment, *n* = 98; BBR, berberine treatment, *n* = 85; Prob + BBR: berberine plus probiotics treatment, *n* = 102; *P < 0.05, **P < 0.01, ***P < 0.001, two-sided Kruskal–Wallis test. Dark lines in the boxes indicate medians, the width of the notches is the IQR, the lowest and highest values within 1.5 times the IQR from the first and third quartiles. **b** Distance-based redundancy analysis (dbRDA) plot based on Bray–Curtis distances of species in post-treatment samples was performed to assess the difference between the four treatment arms (Permanova *P* < 0.001). Projection of species-level gut microbiome samples constrained by treatment methods. Marginal box plots show the separation of the constrained projection coordinates (boxes show medians and quartiles, error bars extend to most the extreme value within 1.5 interquartile ranges), Plac, *n* = 96; Prob, *n* = 98; BBR, *n* = 85; Prob + BBR, *n* = 102. **c** Venn diagram showing the overlapping of microbial species among the four treatment arms that were altered from baseline to post treatment, two-sided Wilcoxon matched-pairs signed-rank test, *q* < 0.05. **d** Heatmap of gut microbial species that showed significantly changed their relative abundances (RAs) post treatment vs. baseline. Plac, *n* = 96; Prob, *n* = 98; BBR, *n* = 85; Prob + BBR: *n* = 102. The changes in nine species in probiotics formula ingested by participants were separately shown below. *q < 0.05, two-sided Wilcoxon match-pairs signed-rank test. The colour key represents the Z score. *Bifidobacterium catenulatum*–*Bpc*, *B. catenulatum–Bifidobacterium pseudocatenulatum complex*. Source data and exact *P*-value are provided in the Source Data file.

Metagenomic analysis with high throughput shotgun sequencing^35^ showed that the alterations in the gut microbiome after 1 week of gentamycin treatment (Supplementary Fig. 1a–d) had recovered to the baseline status after 13 weeks of Plac intervention, regarding to the gene count and α-diversity (Supplementary Fig. 1e–f and Supplementary Data 1, Wilcoxon signed-rank test, P > 0.05). Consistently, principal coordinates analysis (PCoA) revealed that the altered overall gut microbial composition in Plac arm at the species and functional level based on Kyoto Encyclopedia of Genes and Genomes Orthologue (KO) profiles (Supplementary Fig. 1c, d) were largely recovered from gentamycin pretreatment (Supplementary Fig. 1g, h). The reconstitution of the gut microbiome after probiotics treatment was similar to that after Plac treatment (Fig. 2a, b, Supplementary Fig. 1e–h and Supplementary Data 1, Wilcoxon signed-rank test, P > 0.05), except for the enrichment of the ingested probiotics species (Fig. 2d and Supplementary Data 2, Wilcoxon matched-pairs signed-rank test, *q* < 0.05). Thus, Probiotics treatment showed similar effects not only on glycaemic control but also on the resilience of the gut microbiota after gentamycin pretreatment with placebo.

BBR (either alone or with probiotics) treatments, instead, significantly altered the gut microbiome composition after 13 weeks of intervention compared to the Plac treatment (Fig. 2) and to that of the baseline and antibiotic treatment groups (Supplementary Fig. 1e, h), but the BBR and Prob + BBR groups shared similar changes in microbial composition and function (Supplementary Fig. 1g, h and Fig. 2). A total of 78 species changed their relative abundances (RAs) in BBR and Prob + BBR but not in the Plac and Prob groups (Fig.2c, d, baseline vs. post treatment, Wilcoxon matched-pair signed-rank test, *q* < 0.05). Among the 78 BBR-induced species, 36 were designated as the key BBR responsive taxa, the RAs of which showed significant alterations in post-treatment faecal samples of both BBR treatment groups compared to those in Plac or Prob group (Supplementary Fig. 2 and Supplementary Data 3, Dunn’s *P* < 0.05, vs. Plac, or vs. Prob, Kruskal–Wallis (KW) test). BBR depleted the species that mainly produce single sugar or SCFAs from fermenting polysaccharides or oligosaccharides, including *Roseburia* spp., *Ruminococcus bromii*, *Faecalibacterium prausnitzii* and *Bifidobacterium* spp., which were frequently reported to cross-feed with the other saccharides degraders^36–39^. The species enriched by BBR included two *Bacteroides* spp. and multiple taxa of γ-Proteobacteria, which were also induced by metformin treatment^11,15^. Probiotics supplementation did not affect the global alterations in gut microbiome composition induced by BBR (Supplementary Fig. 1g, h and Fig. 2), except for elevating the RAs of probiotics species but not that of *Bifidobacterium longum*.

The pathway enrichment analysis (Supplementary Data 4) showed that compared to the control groups, BBR significantly attenuated protein translation, DNA replication, and fatty acid and amino acid biosynthesis, which was attributed to the bacteriostatic characteristics of BBR. BBR induced the degradation potential of multiple xenobiotics and glycans. BBR also elevated the bacterial response functions similar to metformin^11^, e.g., the bacterial secretion system, the two-component system and the ABC (ATP-binding cassette) transport were promoted. For the most part, Prob + BBR affected similar functional pathways with the BBR group (Supplementary Data 4).

### BBR altered microbial BA metabolism and the blood BA pool

**Fig. 3:**
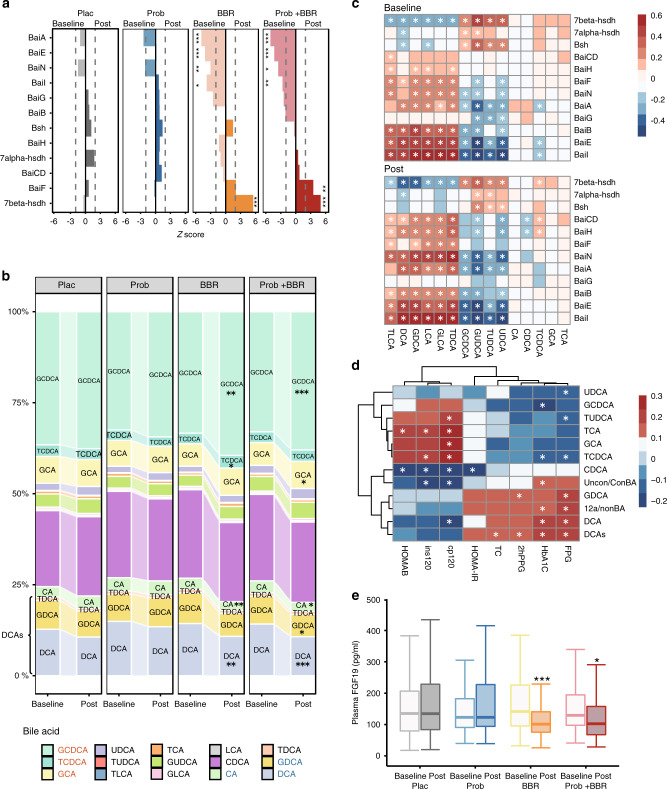
BBR altered microbial BA metabolism and correlated with blood BAs and clinical outcomes. **a** Changes in RAs of bile acid-inducible (Bai) genes induced by the treatments of four arms. hsdh, hydroxysteroid dehydrogenase; *Bsh*, gene encoding bile salt hydrolase. The *Z*-score was calculated with the two-sided Wilcoxon matched-pairs signed-rank test. A *Z*-score > 0 indicated an increase after treatment, while a *z*-score < 0 indicated a decrease after treatment. *P < 0.01, **P < 0.001, ***P < 0.0001; Plac, Placebo, *n* = 96; Prob, probiotics treatment, *n* = 98; BBR, berberine treatment, *n* = 85; Prob + BBR, berberine plus probiotics treatment, *n* = 102. **b** Comparisons of bile acid (BA) composition between baseline and post treatment in the four arms. CA, cholic acid; CDCA, chenodeoxycholic acid; DCA, deoxycholic acid; GCA, glycocholic acid; GCDCA, glycochenodeoxycholic acid; GDCA, glycodeoxycholic acid; GLCA, glycolithocholic acid; GUDCA, glycoursodeoxycholic acid; LCA, lithocholic acid; TCA, taurocholic acid; TCDCA, taurocholic chenodeoxycholic acid; TDCA, taurodeoxycholic acid; TLCA, taurolithocholic acid; TUDCA, tauroursodeoxycholic acid; UDCA, ursodeoxycholic acid. *q < 0.01, **q < 0.001, ***q < 0.0001, two-sided Wilcoxon match-pairs signed-rank test. **c** Correlations between microbial BA genes and blood BA compositions at the baseline (upper panel) vs. post treatment (lower panel) for all participants, Spearman correlation, colour key represented rho value, *q < 0.01. **d** Heatmap of correlations between the blood BAs and clinical outcomes. Multivariate GEE controlling for age, sex and BMI. The colour key represents the *β*-value, *q < 0.01. **e** Plasma FGF19 levels pre and post treatment, *P < 0.05, **P < 0.01, ***P < 0.001, two-sided Wilcoxon matched-pairs signed-rank test, dark lines in the boxes indicate medians, the width of the notches is the IQR, the lowest and highest values within 1.5 times the IQR from the first and third quartiles, Plac, *n* = 96; Prob, *n* = 98; BBR, *n* = 85; Prob + BBR: *n* = 102. 12a/nonBA, 12a-hydroxylated/non–12a-hydroxylated bile acids; 2hPPG, post-load plasma glucose; cp120, post-load serum C peptide; FPG, fasting plasma glucose; HbA1c, glycated haemoglobin; HOMA-IR, homoeostasis model assessment index for assessing insulin resistance; HOMA-β, homoeostasis model assessment index for assessing β-cell function; ins120, post-load serum insulin; TC, total cholesterol; Uncon/Con BA, unconjugated/conjugated bile acids. Baseline, baseline levels; post, post-treatment levels. Source data and exact *P*-value are provided in the Source Data file.

BAs are known to regulate host metabolic homoeostasis and the gut microbiota plays key roles in modulating host BA pool composition, and hence BA signalling^21,22^. Different microbial BAs mediate the therapeutic effects of either acarbose or metformin, the widely prescribed antidiabetic medicines^14,15^. We thus sought to investigate whether microbial BA metabolism and host blood BA pool were also affected by BBR treatment. In addition to depleting the *Eggtherlla lenta* that harbours the complete BA-induced operon (*Bai*)^40,41^ (Fig. 2d and Supplementary Fig. 2), BBR also decreased the total RAs of multiple genes involved in microbial BA metabolism, including *BaiI*, *BaiA*, *BaiN* and particularly the *BaiE* that encodes the rate-limiting enzyme of 7α/β dehydratases, whereas none of these genes showed significant changes in abundance in the Plac or Prob arm (Fig. 3a). Echoed with the changes of *Bai* genes in faeces, the plasma BA profiling by liquid chromatography/mass spectrometry (LC/MS) detected significant increases in glycochenodeoxycholic acid (GCDCA) and decreases in deoxycholic acid species (DCAs), including DCA, glycine and taurine-conjugated DCA (glycodeoxycholic acid and taurodeoxycholic acid (TDCA)) after BBR treatment, contributing to the decreased blood unconjugated/conjugated BA ratio (Uncon/con BA) and secondary BA components (Fig. 3b and Supplementary Data 5). Furthermore, the positive correlation between *Bai* genes RAs and blood secondary BA (DCAs and lithocholic acids (LCAs)) levels were strong and consistent in both baseline and post-treatment measurements, supporting the microbial origins of circulating secondary BAs (Fig. 3c). Thus, although the RAs of *Bsh* were not altered, changes in the blood BA profile suggested that two key gut microbial transformation procedures, the BA deconjugation and dehydroxylation, could be both inhibited by BBR treatment. The GEE analysis in participants from both BBR arms showed that the changes in blood DCAs were significantly correlated with the HbA1c, FPG, PPG and TC improvements, which were the main clinical outcomes of BBR treatment (Fig. 3d), and this relationship was consistent when analysis was performed in the single BBR containing arm (Supplementary Data 6). Of note, similar to the metformin and acarbose results, the plasma FGF19 levels were also reduced in both BBR treatment groups (Fig. 3e). The above results suggested that BBR treatment reduced the gut microbial BA transformation and hence lowered the gut FXR activity, which may contribute to its antidiabetic effect.

### BBR inhibited *R. bromii* to attenuate DCA transformation

**Fig. 4:**
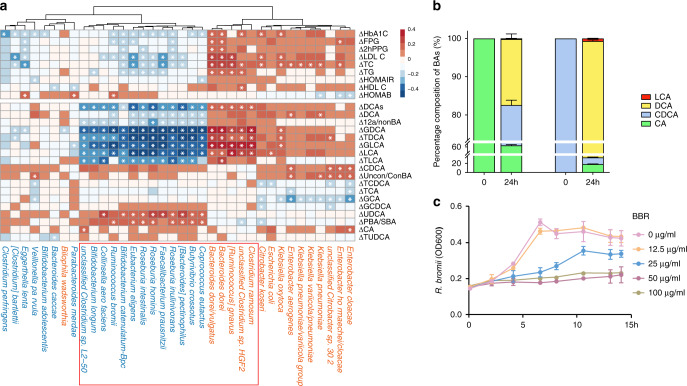
*R. bromii* was inhibited by BBR to attenuate DCA transformation. **a** The two-panel heatmap on the left shows the correlations between the key BBR responsive species and with major clinical outcomes and plasma levels of bile acid. The colour key shows Rho calculated by partial Spearman’s correlation with adjustment for age, sex and BMI. Δ of clinical parameters or BAs = 100% × (baseline value − post treatment value)/baseline value. Species in blue represent depleted species and species in orange represent enriched species after BBR treatments. *P < 0.05. **b** Bile acid transformation assay for *R. bromii*. The percentage composition of deoxycholic acid (DCA) and lithocholic acid (LCA) in the culture media with which *R.bromii* had grown for 24 h with primary bile acid (CA and CDCA) treatment were measured by LC/MS. *n* = 3, data are shown as the mean ± SD. **c** The growth curve of *R. bromii* with different concentrations of BBR in the in vitro culture experiment, demonstrated a significant inhibitory effect of BBR on *R. bromii* starting at a concentration of 25 μg ml^−1^, *n* = 3, *P* < 0.001, determined by two-way repeated-measures ANOVA, data are shown as the mean ± SD. *Bifidobacterium catenulatum* − *Bpc*, *B. catenulatum–Bifidobacterium pseudocatenulatum complex*. Source data and exact *P*-value are provided in the Source Data file.

To determine which commensal bacteria affected by BBR might mediate its inhibitory effect on microbial BA metabolism, we further examined the correlations of the post-treatment RAs of key BBR responsive species (Supplementary Fig. 2) with the changes in clinical outcomes and the changes in plasma BA levels. We found that most secondary BA correlating species were those also associated with the changes of HbA1c and other clinical outcomes, including mainly LDL-C, TC, and TG (Fig. 4a, *P* < 0.05). The HbA1c-correlated taxa were dominated by those that were depleted by BBR treatment including *R. bromii*. Interestingly, most of these taxa are not BA converters, except for *Eggthella lenta*, suggesting the existence of unknown BA metabolism potential in these species. Strains of *Ruminococcus* have been reported to regulate BA metabolism^42,43^ and we thus performed in vitro culture experiments on one strain of *R. bromii*, AF25-7, isolated from a faecal sample of a Chinese woman^44^, to test whether the strain could transform primary BAs. To our surprise, this AF25-7 strain not only demonstrated a substantial DCA transformation ability (Fig. 4b, *P* < 0.001) in vitro but also showed significant growth inhibition in response to BBR at a concentration as low as 25 μg/ml in vitro (Fig. 4c). Thus, *R. bromii* could be the target of BBR in the gut microbiome to reduce the microbial production of secondary BA that is associated with the effective glycaemic control achieved with BBR.

### Probiotics improved glycaemic control in older participants treated with BBR

Subgroup analysis in pre-stratified age (<50 and ≥50 years) groups showed that probiotics, with comparable baseline values, marginally but significantly improved the antidiabetic effect of BBR in participants older than 50 years and exerted the extra benefit of improving HOMA-IR (Supplementary Table 2 and Supplementary Tables 7 and 8). Similar benefits from probiotic supplementation were shown in participants older than 54 years of age (median age in this population) (Supplementary Table 1). Probiotics containing species were significantly more enriched after treatment in older participants than in younger ones and the post-treatment RAs of *Lactobacillus crispatus* and *Lactobacillus salivarius* were only significantly elevated in older participants compared with their baseline RAs (*P* < 0.05, Wilcoxon matched-pairs signed-rank test, Supplementary Fig. 3). Moreover, probiotic containing species, except for *B. longum*, exhibited a dose–response relationship with the improvement in HbA1c levels in the older but not younger participants of the Prob + BBR arm (Supplementary Fig. 4, Spearman correlation, *P* < 0.05). However, the differences of both *R. bromii* and DCAs between the Prob + BBR and BBR arms shown in the older participants were similar with those in the total population (Supplementary Figs. 5 and 6), suggesting that the *R. bromii/*DCAs might not be the cause of the extra benefit of probiotic supplementation in older participants.

## Discussion

In this multicentre, randomized, double-blind, Plac-controlled clinical trial conducted in 409 drug-naive T2D patients, we confirmed the hypoglycaemic effect of BBR in Chinese participants and demonstrated the BBR-induced changes in the human gut microbiome and blood BA pool composition in comparison with the Plac. The triple association between the gut microbiome, blood BAs and clinical outcomes suggested a potential microbial-related mechanism underlying the metabolic benefits of BBR. *R. bromii*, identified as a DCA convertor in this study, might serve as a microbial target of BBR. Our study failed to find significant metabolic improvement with probiotic supplementation in T2D patients, except when it was used in combination with BBR in the older participants.

Probiotics have recently been suggested to delay the recovery of microbiome symbiosis from baseline conditions in healthy volunteers treated with antibiotics^45^. However, this observation implies that interventions following antibiotics pretreatment might bear an opportunity to reset the gut microbiome from the diseased status, such as obesity or T2D-related microbial dysbiosis^4,17^. Potential approaches can include either treatment with beneficial bacteria (replenishment, such as probiotic supplementation) or suppression of the growth of unfavourable taxa with agents such as BBR (surveillance) or both, following temporary antibiotic treatment. However, we did not find a superior effect of Prob compared to that of Plac or Prob + BBR to BBR in treating diabetes, nor were there different changes in gut microbiome symbioses compared to those of Plac. Such findings could be the result of strain-specific functional variation, suggesting the requirement of a more precise strategy for probiotics treatment. This was consistent with the conclusion from preponderant literatures^46–48^ that probiotics have limited effects in the treatment of metabolic diseases. Therefore, the strategy of surveillance, including, e.g., the use of BBR might be more effective than the strategy of replenishment for treating hyperglycaemia in the context that correcting gut microbiota dysbiosis is a feasible and effective way to manage T2D.

Owing to the methodological or population differences, studies on human samples from cohorts taking different antidiabetic medications currently have identified few concordant taxa of so-called antidiabetic bacteria. The microbial BA transformation pathway seems to be targeted by diverse antidiabetic agents, either to decrease 7α/β dehydroxylation or to alter bile salt deconjugation, which consequently modulate the host BA pool and hence mediates the hypoglycaemics effect of medications^11,14,15^. *R. bromii* has been previously reported mainly to ferment dietary carbohydrates and produce single sugar or SCFA, such as acetate, but not propriate or butyrate^39,49^ Consistently, none of the butyrate-producing gene has been identified in multiple *R. bromii* strains isolated from the Chinese population in our previous work^44^. The correlation analysis together with the in vitro BA biotransformation experiment in this study revealed the unprecedently reported DCA production capacity of this species and supported the causal relationship between changes in plasma DCA and the faecal *R. bromii* abundances after BBR treatment. Thus, the *R. bromii*/DCA axis could be one of the gut microbial effectors of BBR with regard to its antidiabetic effect. Regarding the fact that the complete *Bai* operon is not known to be present in *R. bromii* genomes, it is possible that other uncharacterized proteins that regulate microbial BA metabolism might exist in this taxon to regulate the DCA transformation. Further in vivo and in vitro studies are required to delineate the molecular mechanism by which *R. bromii* to produce DCA and how other key BBR responsive species may be involved in the hypoglycaemic effect of BBR.

It is noteworthy that metformin has been reported to increase conjugated UDCA levels, which further attenuated gut FXR activity by inhibiting Bsh activity^15^. However, neither GUDCA nor TUDCA levels were altered after treatment with BBR or Prob + BBR, nor was the RA of *Bsh*. It is possible that the discrepancy between the effects of these two medications on microbial BA metabolism might have resulted from the different target taxa or other BA biotransformation enzymes that were affected by treatments. For instance, the RA of *BaiE*, the rate-limiting enzyme for bacterial secondary BA metabolism was inhibited by BBR but not by metformin. Therefore, the DCA species rather than its upstream UDCAs were altered by BBR. As the most abundant BA component in faeces, the alterations in DCA should have been the main contributor to the fluctuations of gut FXR activity. The downregulated plasma levels of FGF19 further supported our hypothesis that the decrease of DCA species by BBR could diminish the gut FXR. In vivo studies should be employed in the future to confirm this hypothesis.

Notably, the *R. bromii*/DCAs axis was unrelated to the additional benefit of Prob+BBR in reducing HbA1c in older participants. The beneficial effects of probiotics in aged host have been sporadically reported^50,51^. Probiotics exhibit metabolic benefits by improving the gut barrier and alleviating inflammation^52^, which are also key to the development of ageing-related diseases^53^. BBR suppressed multiple *Bifidobacterium* spp. either in the human participants in our study or in rodents^31,54^. Health-associated *Bifidobacterium* spp. have been shown to be depleted along with ageing but enriched in extremely aged healthy subjects^34^. Thus, our probiotics formula containing 2 strains of *Bifidobacterium* might be of particular benefit for older T2D patients treated with BBR. It thus might not be appropriate to connect the potential benefits to the health of aged patients to the add-on hypoglycaemic benefits of supplementing probiotics with BBR, but at least possible that the effect of probiotic supplements on metabolic disorders might be related with the recipient age and the medications.

This study has several limitations. First, as this trial was conducted in Chinese people residing in China and had a relatively short duration for randomized intervention, the findings derived from this investigation may not be generalized to other racial/ethnic populations without caution. Second, the participants enroled in our study were all drug naive with relatively short duration of diabetes and records of lifestyle interventions were not obtained. Our design of the randomized, placebo-controlled, parallel four-arm trial has largely reduced the potential study effects, which might be introduced by unstable metabolic conditions, but future studies should enrol participants with longer disease durations and record detailed lifestyle changes. In addition, more participants experienced gastrointestinal AEs in the BBR-treated groups than that in the Plac or Prob groups, although the AEs did not affect the antidiabetic effect of BBR or gut microbiome features in this study with a 3-month treatment, again the concern needs to be addressed in trials with longer intervention duration. Notwithstanding these limitations, our findings may have important implications for managing T2D in patients by treating microbiome dysbiosis.

## Methods

### Trial design and oversight

We conducted a randomized, double-blind, -placebo-controlled clinical trial in 20 medical centres in China (ClinicalTrials.gov number, NCT02861261). Participants were enroled between 18 August 2016 and 18 July 2017. The trial conformed to the provisions of the Declaration of Helsinki and was approved by the ethics committees at each participating centre. All the participants provided written informed consent.

### Participants and intervention procedure

The eligible participants were those with newly diagnosed T2D according to the World Health Organization criteria^55^ and were drug naive for glycaemic control but with at least 2 months of stable lifestyle intervention.

After completing the screening assessment (from −2 weeks to −3 days), eligible participants were given an oral broad-spectrum antibiotic (gentamicin sulfate 80 mg twice daily) for 7 days during the run-in period, to improve probiotic colonization^34^. Then, the participants were randomly assigned into one of the following four groups in a 1 : 1 : 1 : 1 ratio as follows: BBR (0.6 g per 6 pills, twice daily before meal) plus probiotics (4 g per 2 strips of powder, once daily at bedtime) (Prob + BBR), probiotics plus Plac (Prob), BBR plus Plac (BBR), or Plac plus Plac (Plac). Treatments were administered for 12 weeks and patients visited the centre every 4 weeks until the end of the study. The randomization procedure was stratified by age group and utilized a block size of eight, and the random numbers were generated by utilizing a validated interactive Web-based Response System, which was maintained by an independent data manager. The study personnel and participants were blinded to the assignment of treatment arms.

### The detailed inclusion criteria

Patients are eligible to be included in the study only if they meet all of the following criteria

1. Newly diagnosed T2D according to the 1999 World Health Organization criteria (Appendix 4). Both genders eligible.
2. Age: ≥20 and <70 years.
3. BMI: ≥19.0 and ≤35.0 kg m^−2^.
4. Fully understand the study.
5. Give written informed consent.
6. Are drug naive (have been treated with healthy lifestyle modification only) for management of hyperglycaemia (including oral antidiabetic agents, GLP-1 agonists, or insulin).
7. Have at least 2 months of lifestyle intervention (diet and exercise) for glycaemic control before screening.
8. HbA1c ≥ 6.5% and ≤10.0%, and FPG ≥ 7.0 and ≤13.3 mmol L^−1^ at screening.

### The detailed exclusion criteria

Patients will be excluded from the study if they meet any of the following criteria:

1. Severe liver dysfunction, defined as serum alanine aminotransferase concentration more than 2.5 times above upper limit of normal range. Impaired renal function (defined as serum-creatinine > 132 μmol L^−1^ or estimated glomerular filtration rate (eGFR) < 60 mL (min × 1.73 × m^2^)^−1^); psychiatric disease, severe infection, severe anaemia and neutropenia.
2. Severe organic heart diseases, including but not limited to congenital heart disease, rheumatic heart disease, hypertrophic or dilated cardiomyopathy. New York Heart Association class (NYHA) grade of heart function ≥ III.
3. Allergic to gentamycin or other amino glycosides antibiotics.
4. Type 1 diabetes, monogenic diabetes, diabetes due to injury of the pancreas or other secondary diabetes mellitus (due to such as Cushing syndrome, thyroid abnormalities or acromegaly).
5. Is previously or currently treated with antidiabetic agents, including oral antidiabetic agents, GLP-1 agonists or insulin.
6. Have taken BBR hydrochloride tablets in the past 1 year or previously used BBR hydrochloride tablets for more than a week.
7. Taken other probiotics or probiotics product in the past 3 months.
8. History of acute diabetic complications including diabetic ketoacidosis or hyperosmolar hyperglycaemic non-ketonic coma within 3 months.
9. Taken weight control drugs (including weight-loss drugs); oral, intramuscular, intravenous, non-alimentary canal or intra-articular administration of corticosteroid hormones in the past 3 months.
10. Pregnancy.
11. Participated in other clinical trials in the past 3 months.
12. Medical history of malignant tumour (except local skin basal cell carcinoma) in the past 5 years, whatever with evidence of recurrence or metastasis or not.
13. History of active substance and alcohol abuse. History of alcohol-related diseases in the past 2 years.
14. Having digestive tract disease, which causes accurate and chronic diarrhoea or severe constipation.
15. Medical history of intestine resection or other digestive tract surgery (such as cholecystectomy) in the past 1 year, or other non-gastrointestinal surgery in the past 6 months.
16. Any condition, which in the investigator’s opinion, could interfere with the results of the trial.

### The detailed special criteria for the study of gut microbiome

1. Keep light diet for 3 days before the screening and during the whole study period, avoid fatty foods unless with special requirements.
2. Do not eat fermented dairy products (such as yoghurt) and probiotics for at least 7 days before the screening and during the entire research.
3. Do not take antibiotics (such as penicillin, cephalosporins, tetracycline, etc.) other than the study medication, or other interventions that could affect the gastrointestinal tract for 2 months before the screening and during the whole study period. If antibiotics must be taken for special reasons such as for the patients’ safety consideration by the judgement of the investigators, the use of antibiotic medications must be recorded in detail in the Concomitant Medication Form.
4. Taking steroids, cyclosporine (immunosuppressive agent) or antitumor agents 3 months before the screening and during the whole study period are not permitted.

### The list of institutional review boards

1. Ruijin Hospital Ethics Committee, Shanghai Jiao Tong University School of Medicine, Shanghai, PR China.
2. Ren Ji Hospital Ethics Committee, Shanghai Jiao Tong University School of Medicine, Shanghai, PR China.
3. Shanghai Tenth People’s Hospital Ethics Committee, Tongji University, Shanghai, PR China.
4. Xin Hua Hospital Ethics Committee, Shanghai Jiao Tong University School of Medicine, Shanghai, PR China.
5. Central Hospital Ethics Committee, Minhang district, Shanghai, PR China.
6. Chang Hai Hospital Ethics Committee, Second Military Medical University, Shanghai, PR China.
7. Tong Ren Hospital Ethics Committee, Shanghai Jiao Tong University School of Medicine, Shanghai, PR China.
8. Shanghai First People’s Hospital Ethics Committee, Shanghai Jiao Tong University School of Medicine, Shanghai, PR China.
9. The Second Affiliated Hospital Ethics Committee, Zhejiang University School of Medicine, Zhejiang Province, PR China.
10. The First Affiliated Hospital Ethics Committee, Wenzhou Medical University, Zhejiang Province, PR China.
11. Xuzhou Central Hospital Ethics Committee, Jiangsu Province, PR China.
12. Nanjin Drum Tower Hospital Ethics Committee, Nanjing University Medical School, Jiangsu Province, PR China.
13. Jiangsu Province Hospital Ethics Committee, The First Affiliated Hospital of Nanjing University Medical School, Jiangsu Province, PR China.
14. Qilu Hospital Ethics Committee, Shandong University, Shandong Province, PR China.
15. Peking University Shenzhen Hospital Ethics Committee, Shenzhen, PR China.
16. The First Affiliated Hospital Ethics Committee, Sun Yat-sen University, Guangdong Province, PR China.
17. Sun Yat-sen Memory Hospital Ethics Committee, Sun Yat-sen University, Guangdong Province, PR China.
18. Fujian Provincial Hospital Ethics Committee, Fujian Province, PR China.
19. Wuhan Union Hospital Ethics Committee, Tongji Medical College, Huazhong University of Science and Technology, Hubei Province, PR China.
20. Nanfang Hospital Ethics Committee, Southern Medical University, Guangdong Province, PR China.
21. Institutional Review Board of BGI-Shenzhen, Guangdong Province, PR China.

At baseline and each visit thereafter, questionnaires were completed about patient medical history, acceptability of the study medication, adherence and adverse events. Blood for HbA1c, serum insulin and C peptide levels were determined in a centralized assayed. Other specimens were transported with dry ice to the centre laboratory and stored at −80 °C thereafter.

The clinical outcomes included the improvement of glycaemic control, defined as the changes in HbA1c levels, as the primary outcome and the changes in fasting or post-load blood glucose, lipids, insulin, HOMA-IR and HOMA-β for assessing β-cell function as the secondary outcomes, from baseline to a 13-week follow-up.

BBR used in the present study was produced by industrialized synthesis. The multi-strain probiotics products contained nine proprietary strains of probiotics seen below. BBR, probiotics and their matching Plac were the courtesies from Northeast Pharmaceutical Group Co., Ltd, Shenyang, Liaoning, China, and Shanghai Jiaoda Onlly Co., Ltd, Shanghai, China, respectively. The two companies had no role in the design and conduct of the study; collection, management, analysis or interpretation of the data; preparation, review or approval of the manuscript; or decision to submit the manuscript for publication.

A CONSORT checklist of information reporting a randomized trial was included (Supplementary Note 1).

### Biochemical measures

HbA1c, serum insulin and C peptide were performed in central laboratory in Ruijin Hospital. HbA1c was measured by high-performance liquid chromatography using the VARIANT II Haemoglobin Testing System (Bio-Rad Laboratories, Hercules, CA, USA). Serum insulin and C peptide were measured by electrocheuminescence immunoassay “ECLIA” on cobase601 immunoassay analysers (Roche Diagnostic, Basel, Switzerland). The sensitivity range, intra-assay coefficient of variability (CV) and inter-assay CV for HbA1c were 3.5–19.0%, 0.39 and 0.45; for insulin was 0.2–1000 μIU mL^−1^, 1.1 and 3.6; C-peptide was 0.01–40.0 ng mL^−1^, 0.7 and 1.95, respectively.

### Metagenomic analysis

For metagenomic library construction and sequencing, we used the BGISEQ-500 platform as previously described^35^. In brief, DNA samples were subjected to random fragmentation, end-repair and subsequent adaptor ligation for DNA nanoball-based library construction and combined primer anchor synthesis-based shotgun metagenomic sequencing using a paired-end 100 bp mode. A total of 1192 faecal DNA samples, from three time points (baseline, *n* = 405; after 1 week of antibiotic treatment, *n* = 403; and after four-arm-based 3-month interventions, *n* = 384) were sequenced and subjected to subsequent metagenomic analysis. After removing low-quality and human-derived sequences as described^35^, high-quality non-human reads (9.98 ± 2.31 GB per sample) were aligned to the 9.9 M integrated gene catalogue (IGC) by SOAP2.22 using the criterion of ≥90% identity. Sequence-based gene abundance profiling was performed as follow^56^.

Step 1: For any sample S, calculation of the copy number of each gene:

$$b_i = \frac{{x_i}}{{L_i}}$$

Step 2: Calculation of the RA of gene *i*:

$$r_i = \frac{{b_i}}{{\mathop {\sum }\nolimits_j b_j}}$$

*r*_*i*_: the RA of gene *i* in sample S.

*x*_*i*_: the number of mapped reads.

*L*_*i*_: the length of gene *i*. The RAs of phyla, species and KOs were calculated by the sum of the RAs of their annotated genes. The number of genes, which represented gene richness was calculated for each sample in accordance with a previous study^56^. Alpha diversity was quantified by the Shannon index using RA profiles at the gene level. At the species level, we further confined our analyses to species with at least 100 annotated genes in each of at least 20% of samples, which resulted in 131 species accounting for on average 99.56% of the annotated microbial species composition. Except for *B. longum*, eight of the nine probiotics containing species including *Bifidobacterium breve*, *Lactobacillus casei*, *L. crispatus*, *Lactobacillus fermentum*, *Lactobacillus plantarum*, *Lactobacillus rhamnosus*, *L. salivarius* and *Lactobacillus gasseri* did not meet the above selection criteria, and thus they were subjected to further analyses separately from the profiling of the 131 species.

Gut microbial dissimilarities between groups at the species and KO level were visualized by unconstrained PCoA, using Bray–Curtis dissimilarities based on species and KO profiles (PCoA function, R 3.3.2, ape package). Distance-based redundancy analysis between four treatment arms was also conducted using the RAs of species (capscale function, R 3.3.2, vegan package).

Differentially enriched the Kyoto Encyclopedia of Genes and Genomes (KEGG) pathways (modules) between groups were identified according to the reporter *Z*-scores of all detected KOs involved in the given pathway (module)^57^. An absolute reporter score value ≥ 1.96 (95% confidence according to normal distribution) was used as the detection threshold for significance.

The institutional review board of BGI-Shenzhen approved the analyses of faecal samples/meta data collected by all participating centres under ethical clearance number BGI-R087-1-T1.

### Profiling of microbial genes involved in BA biotransformation

The identification of microbial genes involved in BA biotransformation was performed as previously described^14^. According to analysis with the updated KEGG database (Version 87), in the secondary BA (SBA) biosynthesis pathway (map00121), a baiN gene encoding enzymes (K07007, 3-dehydro-bile acid Delta4,6-reductase [EC: 1.3.1.114]) involved in the final steps of SBA biosynthesis was newly recruited in this study. The RA of each BA gene was also calculated from the sum of their annotated genes.

### Metabolomic measures

A total of 746 plasma samples from baseline and post-treatment collections (Plac *n* = 96, Prob *n* = 96, BBR *n* = 81 and Prob + BBR *n* = 100) were subjected to the blood BA profile analysis, covering over 15 BA species. Sample preparation as described in previous study^14^. An extraction solvent was made with methanol containing 0.1 μg mL^−^^1^ cholic acid (CA)-d4, 0.3 μg mL^−1^ chenodeoxycholic acid (CDCA)-d4, 0.2 μg mL^−1^ glycocholic acid-d5, 0.2 μg mL^−1^ GCDCA-d4, 0.1 μg mL^−1^ taurocholic acid-d5 and 0.1 μg mL^−1^ TDCA-d5. Quality-control samples made from a mixture of equal volume of all serum samples were prepared in the same method as the serum samples and were analysed once after every ten real samples.

A Vanquish UPLC-Q Exactive (Thermo Fisher Scientific, Rockford, IL, USA) and an ACQUITY UPLC HSS T3 column (100 mm × 2.1 mm, 1.8 μm, Waters, Milford, MA, USA) were used for LC separation. The oven temperature was 50 °C and the flow rate was 0.35 mL min^−1^. A 7e4 resolution MS full scan mode with a scan range of m z^−1^ 80–1200 was used in the analysis. The spray voltage was 3.5 kV for positive mode and 3.00 kV for negative mode. The capillary temperature was 300 °C and the auxiliary gas heater temperature was 350 °C. The sheath gas and auxiliary gas were 45 and 10 (in arbitrary units), respectively.

Plasma FGF19 (R&D Systems, Minneapolis, MN, USA) was analysed using commercially available enzyme-linked immunosorbent assay kits in accordance with the manufacturer’s instructions.

### Multi-strain probiotics composition

The multi-strain probiotics consists of nine proprietary strains of lactic acid bacteria. Each sachet contains ≥50 billion colony forming unit (CFU) of live, freeze-dried bacteria (Supplementary Table 9).

### Growth experiment of *R. bromii*

*R. bromii* strain number AF25-7 was isolated from faecal sample of a healthy Chinese adult^44^. It was cultured in MPYG medium (Supplementary Table 10) and incubated in anaerobe chamber, BACTRON600-28 (SHELLAB, Cornelius, OR, USA) with 5% hydrogen, 10% carbon dioxide and 85% nitrogen at 37 °C. The 16S rRNA gene of *R. bromii* was amplified by the PCR and sequenced, to ensure the successful recovery of *R. bromii* from −70 °C. The primers used for 16S rRNA gene were: 341 F: 5′-CCTACGGGAGGCAGCAG-3′, 926 R: 5′-CCGTCAATTCCTTTRAGTTT-3′. For growth curve experiment, we seeded *R. bromii* at 10% in a volume of 1.5 ml media with different concentration of BBR (0, 12.5, 25, 50, 100 μg ml^−1^) and measured OD600 of the bacterial culture every 1–2 h in a plate reader (CMax Plus, Molecular Devices, San Jose, CA, USA). Six replicates were prepared in three independent experiments. Growth curve and BA biotransformation of in vitro culture experiment were assessed with two-way analysis of variance (ANOVA) and unpaired Student’s *t*-test; *P*-values reported were two-sided; statistical significance was defined as *P* < 0.05.

### In vitro BA biotransformation of *R. bromii*

The BA transformation assay was performed at an independent batch of culture. *R. bromii* was added into 1.5 ml of MPYG medium containing CDCA and CA at an initial concentration of 100 μM and cultured overnight. Vehicle controls were prepared as CDCA containing MPYG medium without adding *R. bromii*. Three replicates were prepared in three independent experiments. Cell-free supernatants were obtained by centrifugation at 12,000 × *g* for 5 min. Quantification of BA in *R. bromii* supernatants was performed on an Acquity H-class UPLC system using a BEH C18 column (Waters) coupled to QTRAP 5500 (SCIEX, Canada) in MRM (multiple-reaction monitoring) mode^58^. BA standards CDCA, CA, DCA and LCA (Sigma-Aldrich) were prepared with distilled water at a final concentration of 100 μM. Stock solutions of the CDCA, CA, DCA and LCA were further diluted with 50% methanol to give final concentrations of 2 to 2000 p.p.b. A mixed-standard solution containing each of the D4-labelled BAs was used as the internal standard solution and added in calibration curves and samples for normalization. SkylineV4.2^59^ was used for data analysis and sample quantification.

### Statistical analyses for clinical parameters

For all studied participants, the aim of the study is a comparison of slopes in repeated measurements with equal allocation among the four treatment arms. Based on previous studies^27,60^, with a sample size of 360 studied participants, the power for the primary outcome reaches 86% (two-sided test, *α* = 5%). We assumed that the overall dropout rate during the study period would be 10%. To account for follow-up losses, the power for the primary outcome was set to 86% if 400 study subjects were recruited.

Statistical analyses of clinical data were performed using SAS version 9.4 (SAS Institute, Cary, NC, USA). All *P*-values reported were two-sided. Data analyses were implemented using intention-to-treat principles based on randomized treatment assignments in which all available data were used and missing data were not imputed, because the rate of participants lost to follow-up was <5% overall. Baseline demographic and clinical characteristics were assessed and compared by treatment group with the *χ*^2^-tests for categorical variables and with ANOVA. For the primary outcome, changes in HbA1c, an analysis of covariance model adjusted for age group (<50 and ≥50 years) was used to examine the difference between treatment groups. The overall difference among the four treatment groups was compared with the use of a global test of unordered groups. If the difference was significant at a *P*-value of <0.05, then all (six) pairwise comparisons were made with adjustments for multiplicity in which statistical significance was defined as *P* < 0.008 after Bonferroni correction.

Multivariate GEE model was used to examine whether Prob or BBR intervention lowered HbA1c levels, as well as other secondary outcomes compared with Plac group and to examine whether Prob + BBR treatment was associated with a significantly lower HbA1c level as compared with Plac group, than Prob or BBR intervention compared with Plac group after controlling for potential confounding factors, including baseline HbA1c level, age, body mass index (BMI), total protein, aspartate transaminase, LDL cholesterol and HOMA-IR.

### Statistical analyses for metagenomics, BAs and their correlations with clinical parameters

Wilcoxon signed-rank tests were applied to detect differences in the gut microbial features (richness, diversity, RAs of species) and plasma BAs levels between baseline and post treatment measurements in each treatment arm. KW tests were applied to detect differences in the gut microbial features (richness, diversity, RAs of species and KOs) between the four groups. Dunn’s post hoc tests were further performed to explore the differences between two groups. A Dunn’s *P*-value < 0.05 was considered significant. The Benjamini–Hochberg (BH) method was used to correct the multiple comparisons of species, genes and blood BAs (function *p.adjust*, package *stats*). A BH-adjusted *P*-value (*q*) < 0.05 was considered significant.

The correlations between the RAs of microbial genes involved in BA biotransformation and plasma BA species were assessed by Spearman’s correlation analysis. The correlations between RAs of microbial species in BBR treatment arms post treatment and changes in (1) BA species and in (2) clinical parameters were assessed by partial Spearman’s correlation analysis after adjustment for age, sex and BMI, and a *P*-value of <0.01 or <0.05 was considered significant, respectively.

GEE analysis was performed to assess the longitudinal associations between changes in BA species and clinical parameters in four treatment arms after adjustment for age, sex and BMI. The *P*-value of each regression coefficient was calculated and a *P*-value of <0.01 was considered significant.

### Reporting summary

Further information on research design is available in the Nature Research Reporting Summary linked to this article.

## Supplementary information

Supplementary Information

Description of Additional Supplementary Files

Supplmentary Data 1

Supplmentary Data 2

Supplmentary Data 3

Supplmentary Data 4

Supplmentary Data 5

Supplmentary Data 6

Supplementary Data 7

Reporting Summary

## Data Availability

Metagenomic sequencing data for the 1192 faecal samples can be accessed from the China Nucleotide Sequence Archive (CNSA) with the dataset identifier CNP0000478 and the National Centre for Biotechnology Information BioProject Database with the dataset accession number PRJNA643353. The metabolomics raw data was shown in Supplementary Data 7. The other datasets analysed in this study were available at KEGG Release 87.0 (https://www.genome.jp/kegg-bin/) and at IGC (http://meta.genomics.cn/meta/dataTools). All other data are available upon request. The source data underlying Figs. 2, 3 and 4, and Supplementary Figs. 1, 2, 3, 4, 5 and 6 are provided as a source data file. The study was approved by Chinese Ministry of Science and Technology (MOST) for the Review and Approval of Human Genetic Resources (approval number 2020BAT0223). Source Data are provided with this paper.

## Source data

Source Data
