# Supplementary material for: Gut microbiome-related effects of berberine and probiotics on type 2 diabetes (the PREMOTE study)
Source: Nat Commun. 2020 Oct 6;11:5015. doi: 10.1038/s41467-020-18414-8 (PMC7538905; doi:10.1038/s41467-020-18414-8)
Supplement: Supplementary file 1 — Supplementary Information [file 41467_2020_18414_MOESM1_ESM.docx]

**Supplementary Materials**

**Gut microbiome related effects of berberine and probiotics on type 2 diabetes (the PREMOTE Study)**

**Zhang et.al.**

**Table of contents**

**Supplementary Table**

Supplementary Table 1 2

Supplementary Table 2 3

Supplementary Table 3 4

Supplementary Table 4 5

Supplementary Table 5 6

Supplementary Table 6 7

Supplementary Table 7 8

Supplementary Table 8 9

Supplementary Table 9 10

Supplementary Table 10 11

**Supplementary Figure**

Supplementary Figure 1 12

Supplementary Figure 2 14

Supplementary Figure 3 15

Supplementary Figure 4 17

Supplementary Figure 5 18

Supplementary Figure 6 19

Supplementary References 19

Supplementary Table 1. Primary Outcome in All Participants and the older participants based on Generalized Estimating Equation Analysis

|  | Model 1 | | | | | |  | | Model 2 | | | | |
| --- | --- | --- | --- | --- | --- | --- | --- | --- | --- | --- | --- | --- | --- |
|  | β (95%CI) | | | †*P* | | β (95%CI) | ‡*P* | | β (95%CI) | | †*P* | β (95%CI) | ‡*P* |
| Total | | | | | | | | | | | | | |
| Plac |  |  |  | |  |  | |  | |  | | |  |
| Prob | 0.03 (-0.08, 0.13) | | | 0.64 | | / | / | | 0.01 (-0.10, 0.12) | | 0.83 | / | / |
| BBR | -0.22 (-0.32, -0.12) | | | 3.12E-05 | | / | / | | -0.21 (-0.32, -0.10) | | 1.07E-04 | / | / |
| Prob +BBR | -0.26 (-0.36, -0.16) | | | 2.15E-07 | | -0.04 (-0.13–0.05) | 0.37 | | -0.25 (-0.35, -0.15) | | 8.72E-07 | -0.05 (-0.14, 0.04) | 0.28 |
| Age ≥50 years | | | | | | | | | | | | | |
| Plac |  | | |  | |  |  | |  | |  |  |  |
| Prob | -0.005(-0.13, 0.12) | | | 0.94 | | / | / | | -0.02 (-0.15, 0.11) | | 0.77 | / | / |
| BBR | -0.17 (-0.31, -0.03) | | | 0.02 | | / | / | | -0.15 (-0.29, -0.01) | | 0.04 | / | / |
| Prob+BBR | -0.25 ( -0.38, -0.12) | | | 1.38E-04 | | -0.08（-0.18, 0.02） | 0.13 | | -0.25 (-0.38, -0.13) | | 9.68E-05 | -0.11 (-0.21, -0.02) | 0.03 |
| Age ≥ 54 years | | | | | | | | | | | | | |
| Plac |  | | |  | |  |  | |  | |  |  |  |
| Prob | -0.04 (-0.18, 0.09) | | | 0.53 | | / | / | | -0.07 (-0.21, 0.07) | | 0.33 | / | / |
| BBR | -0.14 (-0.29, 0.01) | | | 0.08 | | / | / | | -0.12 (-0.28, 0.03) | | 0.12 | / | / |
| Prob +BBR | -0.26 (-0.40, -0.11) | | | 4.12E-04 | | -0.11 (-0.24–0.01) | 0.07 | | -0.27 (-0.41, -0.12) | | 2.36E-04 | -0.15 (-0.27, -0.03) | 0.02 |

BBR, berberine treatment; Prob, Probiotics treatment; Plac, Placebo, Prob+BBR: berberine plus probiotics treatment

Model 1: Generalized estimating equation (GEE) analysis was performed to compare the HbA1c between groups controlling for baseline HbA1c on the basis of intention-to-treat (ITT) analysis;

Model 2: Multivariate GEE analysis was performed to compare the HbA1c between groups controlling for age,

β, Regression coefficient BMI, baseline HbA1c, HOMA-IR, LDL-cholesterol, AST, aspartate transaminase, TP, total protein on the basis of ITT analysis; Abbreviations: BBR, berberine, HbA1c, glycated haemoglobin.

†*P* values refer to comparison between Plac group and the other groups after intervention using GEE model (two-sided wald test).

‡*P* values refer to comparison between Prob+BBR group and BBR alone after intervention using GEE model (two-sided wald test).

Supplementary Table 2. Clinical Characteristics after Treatments in All Participants.

|  | **Plac (n=97)** | | **Prob (n=100)** | | | **BBR (n=90)** | | | **Prob+BBR (n=104)** | | |
| --- | --- | --- | --- | --- | --- | --- | --- | --- | --- | --- | --- |
|  | **13 w** | **Change¶** | **13 w** | **Change¶** | **P** | **13 w** | **Change¶** | **P** | **13 w** | **Change¶** | **P** |
| BW (kg) | 70.4±12.3 | -1.91 (-2.38, -1.44) | 70.3±11.4 | -1.70 (-2.16, -1.24) | 0.60 | 69.4±13.4 | -2.11 (-2.59, -1.63) | 0.53 | 68.9±11.1 | -1.86 (-2.32, -1.41) | 0.94 |
| BMI (kg m^-2^) | 25.6±3.36 | -0.71 (-0.88, -0.54) | 25.0±2.87 | -0.60 (-0.77, -0.43) | 0.64 | 25.0±3.39 | -0.78 (-0.96, -0.61) | 0.42 | 24.8±2.78 | -0.69 (-0.85, -0.52) | 0.76 |
| WC (cm) | 89.8±8.4 | -2.26 (-3.00, -1.53) | 89.8±8.2 | -1.81 (-2.53, -1.09) | 0.45 | 88.1±9.3 | -2.72 (-3.48, -1.96) | 0.24 | 88.4±7.6 | -2.16 (-2.87, -1.45) | 0.81 |
| SBP (mmHg) | 125.9±12.2 | -3.42 (-5.94, -0.91) | 124.5±11.6 | -4.22 (-6.69, -1.75) | 0.43 | 127.1±13.2 | -1.88 (-4.48, 0.71) | 0.41 | 124.4±11.2 | -1.38 (-3.80, -1.05) | 0.89 |
| DBP (mmHg) | 76.5±9.1 | -3.64 (-5.43, -1.85) | 76.3±8.2 | -4.29 (-6.06, -2.52) | 0.61 | 77.7±9.8 | -2 (-3.85, -0.15) | 0.25 | 76.6±8.8 | -2.10 (-3.83, -0.36) | 0.50 |
| FPG (mmol L^-1^) | 7.62±1.95 | -0.52 (-0.77, -0.27) | 7.85±1.46 | -0.55 (-0.80, -0.30) | 0.77 | 6.90±1.29 | -1.26 (-1.52, -1.00) | 9.87E-05 | 6.74±1.21 | -1.33 (-1.58, -1.09) | 7.34E-07 |
| 2hPPG (mmolL L^-1^) | 14.13±3.54 | -0.89 (-1.52, -0.25) | 13.80±3.20 | -1.00 (-1.63, -0.37) | 0.54 | 11.37±2.95 | -2.85 (-3.51, -2.20) | 4.53E-09 | 11.22±2.62 | -3.20 (-3.81, -2.58) | 4.83E-12 |
| Fins (μIUml L^-1^) | 10.82(6.75-16.20) | -2.22 (-3.70, -0.73) | 10.15(7.44-14.92) | -0.66(-2.11, 0.80) | 0.39 | 10.80(7.42-14.53) | -1.57 (-3.11,-0.03) | 0.69 | 8.44 (6.56-12.74) | -2.60 (-4.03, -1.17) | 0.28 |
| ins120 (μIUml L^-1^) | 54.35(40.28-86.69) | 3.26 (-2.13, 8.64) | 47.41(31.49-62.85) | 1.89 (-3.44, 7.21) | 0.29 | 51.28(33.34-81.6) | 0.11(-5.48, 5.70) | 0.68 | 46.78(34.25-77.55) | 3.10 (-2.13, 8.32) | 0.86 |
| FCP (ngml L^-1^) | 2.44(2.02-3.25) | -0.29 (-0.45, -0.13) | 2.41(2.01-3.15) | -0.15 (-0.31, 0.01) | 0.76 | 2.58(1.92-2.95) | -0.16 (-0.32, 0.01) | 0.98 | 2.33(1.89-2.95) | -0.25(-0.41, -0.10) | 0.43 |
| cp120 (ng ml^-1^) | 7.98(6.36-9.52) | 0.26 (-0.10, 0.62) | 6.67(5.64-8.81) | 0.05 (-0.31, 0.40) | 0.07 | 7.91(6.31-9.66) | 0.41 (0.04, 0.79) | 0.72 | 7.53(6.08-10.01) | 0.45 (0.10, 0.80) | 0.70 |
| TG (mmol L^-1^) | 1.49(1.04-1.89) | -0.09 (-0.34, 0.17) | 1.73(1.00-2.70) | 0.25 (0.002, 0.50) | 0.04 | 1.24(0.96-1.81) | -0.41 (-0.67, -0.15) | 3.20E-03 | 1.33 (1.01-1.98) | -0.48 (-0.73, -0.24) | 1.53E-03 |
| TC(mmol L^-1^) | 4.97±0.96 | -0.20 (-0.37, -0.03) | 5.16±1.12 | -0.09 (-0.26, 0.07) | 0.19 | 4.44±1.01 | -0.55 (-0.72, -0.37) | 1.03E-04 | 4.51±0.92 | -0.74(-0.90, -0.58) | 5.04E-08 |
| HDL(mmol L^-1^) | 1.24±0.30 | 0.01 (-0.02, 0.05) | 1.18±0.27 | -0.02 (-0.06, 0.01) | 0.05 | 1.19±0.26 | -0.03 (-0.06, 0.01) | 0.04 | 1.16±0.23 | -0.03 (-0.06, 0.003) | 0.01 |
| LDL (mmol L^-1^) | 3.20±0.87 | -0.14 (-0.28, -0.001) | 3.20±0.87 | -0.24 (-0.37, -0.10) | 0.47 | 2.78±0.82 | -0.45 (-0.59, -0.31) | 1.61E-04 | 2.84±0.77 | -0.56(-0.69, -0.43) | 1.96E-06 |
| HOMA-IR (μIU·mol L^-2^) | 3.59(2.30-5.40) | -1.15 (-1.72, -0.59) | 3.63(2.33-5.42) | -0.50 (-1.04, -0.05) | 0.33 | 3.30(2.12-4.44) | -1.12 (-1.70, -0.54) | 0.36 | 2.59 (1.92-3.84) | -1.28 (-1.82, -0.74) | 0.01 |
| HOMA-ß (IU·mol^-1^) | 54.28 (31.68-99.46) | 5.48 (-2.81, 13.77) | 46.39(36.10-71.63) | 4.36 (-3.72, 12.44) | 0.96 | 65.3(43.97-94.61) | 13.56 (4.99, 22.13) | 7.57E-03 | 59.60(38.90-87.49) | 11.29 (3.29, 19.29) | 0.02 |

BBR, berberine treatment; Prob, Probiotics treatment; Plac, Placebo; Prob+BBR: berberine plus probiotics treatment; BW: Body weight; BMI: Body mass index is the weight in kilograms divided by the square of the height in meter; WC: Waist circumference; SBP: Systolic blood pressure; DBP: Diastolic blood pressure; FPG: Fasting plasma glucose; 2hPPG: Post load plasma glucose; Fins: Fasting serum insuli; ins120: Post load serum insulin; Fcp: Fasting serum C peptide; cp120: Postload serum C peptide; TG: Triglyceride; TC: Total cholesterol; HDL: HDL cholesterol; LDL: LDL cholesterol; HOMA-IR: (Fasting serum insulin * Fasting plasma glucose)/22.5, homeostasis model assessment index for assessing insulin resistance; HOMA- ß: (20 * Fasting serum insulin)/(Fasting plasma glucose - 3.5), homeostasis model assessment index for assessing ß cell function.

For the secondary outcomes, improvements of HOMA-IR compared to placebo group were only observed in Prob+BBR group, but not in BBR-alone group. ¶The values are least-squares means represent changes from baseline (95%CI). Data was presented as Mean±SD or Median (IQR).

P values refer to comparison between each treatment group and placebo group using GEE model after treatment adjusted for baseline values and age (two-sided wald test).

Supplementary Table 3. Adverse Events

| **No** | **Plac (n=103)** | **Prob (n=102)** | †**P** | **BBR (n=98)** | †**P** | **Prob+BBR(n=106)** | †**P** | ‡**P** | **Total (n=409)** |
| --- | --- | --- | --- | --- | --- | --- | --- | --- | --- |
| Total No. of AE | 45 | 29 | 0.02 | 37 | 0.39 | 49 | 0.71 | 0.22 | 160 |
| No. of Gastrointestinal AE | 4 | 6 | 0.51 | 14 | 9.83E-03 | 21 | 3.89E-04 | 0.30 | 45 |
| No. of Hypoglycemia episode | 0 | 1 | 0.31 | 0 | / | 1 | 0.32 | 0.34 | 2 |
| No. of SAE | 2 | 0 | 0.16 | 0 | 0.17 | 1 | 0.54 | 0.34 | 3 |

BBR, berberine treatment; Prob, Probiotics treatment; Plac, Placebo, Prob+BBR: berberine plus probiotics treatment

Abbreviation AE, adverse events; SAE, serious adverse events.

†P values refer to comparison each treatment group and Plac group using two-sided χ2 test.

‡P values refer to comparison between Prob+BBR and BBR group using two-sided χ2 test.

| **Characteristics** | **Plac (n=103)** | | **Prob (n=102)** | | | **BBR (n=98)** | | | **Prob+BBR (n=106)** | | |
| --- | --- | --- | --- | --- | --- | --- | --- | --- | --- | --- | --- |
|  | **0 w** | **13 w** | **0 w** | **13 w** | **P** | **0 w** | **13 w** | **P** | **0 w** | **13 w** | **P** |
| AST(IQR) IU L^-1^ | 22.0 (18**.**0-29.0) | 20.0(16.3-26.0) | 21.0(17.0-27.0) | 20.1(16.0-24.0) | 0.64 | 22.4(18.0-29.0) | 19.1(17.0-25.0) | 0.97 | 21.0 (17.0-26.0) | 18.1(16.0-21.5) | 0.13 |
| ALT(IQR) IU L^-1^ | 28.0 (19.9-43.0) | 22.0(17.0-31.0) | 27.2 (18.0-38.0) | 22.0(17.0-30.0) | 0.39 | 26.0(17.0-36.0) | 21.0(14.0-29.0) | 0.62 | 24.0 (17.0-35.0) | 21.0(14.0-26.0) | 0.45 |
| BUN mmol L^-1^ | 5.40±1.46 | 5.20±1.26 | 5.26±1.27 | 5.54±1.27 | 0.01 | 5.30±1.08 | 5.37±1.13 | 0.10 | 5.20±1.39 | 5.26±1.28 | 0.27 |
| Cr μmol L^-1^ | 65.86±15.77 | 65.99±13.71 | 65.04±16.06 | 65.46±16.70 | 0.50 | 64.89±13.64 | 64.15±14.10 | 0.51 | 64.55±15.48 | 4.09±14.19 | 0.53 |
| TP g L^-1^ | 73.92±4.75 | 73.48±4.79 | 73.64±4.21 | 73.29±4.05 | 0.99 | 73.60±4.50 | 72.88±4.70 | 0.68 | 72.87±4.92 | 72.86±4.03 | 0.90 |
| UA μmol L^-1^ | 330.22±78.90 | 336.84±86.73 | 326.10±86.39 | 331.34±87.94 | 0.62 | 318.46±78.41 | 329.09±82.83 | 0.85 | 331.20±77.32 | 332.78±77.05 | 0.47 |

Supplementary Table 4. Biochemical Measurements

BBR, berberine treatment; Prob, Probiotics treatment; Plac, Placebo, Prob+BBR: berberine plus probiotics treatment

AST,aspartate transaminase; ALT, alanine aminotransferase; BUN, blood urea nitrogen; Cr, creatinine, TP, total protein. Data was present Mean±SD or Median (IQR)

P values refer to comparison between each treatment group and Plac group using GEE model on the basis of intention-to-treat analysis (two-sided wald test). All analyses adjusted for age and baseline values.

Supplementary Table 5. Changes in HbA1c in Participants with Diabetes Duration ≤12 or >12 months, and with or without Gastrointestinal Adverse Events.

|  |  | **Plac (n=97)** |  |  | **Prob (n=100)** |  |  | **BBR (n=90)** |  |  | **Prob+BBR (n=104)** |  |  |
| --- | --- | --- | --- | --- | --- | --- | --- | --- | --- | --- | --- | --- | --- |
|  | **N.** | **Change¶** | †**P** | **N.** | **Change¶** | †**P** | **N.** | **Change¶** | †**P** | **N** | **Change¶** | †**P** | **‡P** |
| **Diabetes duration** |  |  |  |  |  |  |  |  |  |  |  |  |  |
| ≤12 months | 85 | -0.58 (-0.77, -0.39) | 0.68 | 89 | -0.56 (-0.71, -0.40) | 0.24 | 81 | -0.97 (-1.14, -0.80) | 0.34 | 96 | -1.05 (-1.21, -0.88) | 0.71 | 5.44E-06 |
| >12 months | 12 | -0.69 (-1.19, -0.19) |  | 11 | -0.28 (-0.72, 0.15) |  | 9 | -1.22 (-1.72, -0.72) |  | 8 | -0.94 (-1.50, -0.38) |  | 0.09 |
| **The GI adverse events** |  |  |  |  |  |  |  |  |  |  |  |  |  |
| Yes | 4 | 0.05 (-0.81, 0.91) | 0.13 | 6 | -0.93 (-1.52, -0.35) | 0.16 | 11 | -0.87 (-1.33, -0.42) | 0.57 | 17 | -1.00 (-1.38, -0.62) | 0.83 | 0.15 |
| No | 93 | -0.62 (-0.80, -0.44) |  | 94 | -0.50 (-0.65, -0.35) |  | 79 | -1.01 (-1.18, -0.84) |  | 87 | -1.04 (-1.21, -0.88) |  | 8.32E-07 |

BBR, berberine treatment; Prob, Probiotics treatment; Plac, Placebo, Prob+BBR: berberine plus probiotics treatment, GI: Gastrointestinal.

¶The values are least-squares means represent changes from baseline (95%CI). Analysis of variance (ANOVA) were performed to compare the Change in HbA1c between groups.

*P values refer to comparison between groups diabetes duration ≤12 months and >12 months, with and without GI adverse events (ANOVA, two-sided).

**‡**P values refer to comparison between four intervention groups (ANOVA, two-sided).

Supplementary Table 6. All Pairwise Comparisons of HbA1c by Treatment Regimen with Diabetes Duration ≤12 months and without Gastrointestinal Adverse Events.

|  |  | **Participants Diabetes duration ≤12 months** | | | |  | **Participants without GI adverse events** | | | |
| --- | --- | --- | --- | --- | --- | --- | --- | --- | --- | --- |
|  | **N.** | **Change in HbA1c (95%CI)** ¶ | †P | ‡P | **^§^**P | **N.** | **Change in HbA1c (95%CI) ¶** | †P | ‡P | **^§^**P |
| **Plac** | 85 | -0.58 (-0.75, -0.41) | / | / | / | 93 | -0.62 (-0.78, -0.46) | / | / | / |
| **Prob** | 89 | -0.56 (-0.72, -0.39) | 0.83 | 6.92E-04 | / | 94 | -0.50 (-0.66, -0.34) | 0.28 | 2.54E-05 | / |
| **BBR** | 81 | -0.97 (-1.14, -0.80) | 1.65E-03 | / | / | 79 | -1.01 (-1.19, -0.84) | 1.35E-03 | / | / |
| **Prob+BBR** | 96 | -1.05 (-1.20, -0.89) | 9.05E-05 | 0.52 | 2.93E-05 | 87 | -1.04 (-1.21, -0.88) | 3.66E-04 | 0.78 | 4.41E-06 |

BBR, berberine treatment; Prob, Probiotics treatment; Plac, Placebo, Prob+BBR: berberine plus probiotics treatment,GI: Gastrointestinal.

The values are least-squares means, statistical significance was defined as P<0.008 applied after Bonferroni correction.

†P values refer to comparison of change in HbA1c between Plac group and the other groups using ANOVA (two-sided).

‡P values refer to comparison of change in HbA1c between BBR group and the other groups using ANOVA (two-sided) .

**^§^**P values refer to comparison of change in HbA1c between Prob group and the other groups using ANOVA (two-sided) .

Supplementary Table 7. Baseline Characteristics in Subgroups（Age ≥50yrs）

|  | **Plac (n=64)** | **Prob (n=61)** | **BBR (n=61)** | **Prob+BBR (n=72)** |
| --- | --- | --- | --- | --- |
| Age (yr) | 58.8±5.4 | 58.6±4.6 | 59.3±6.0 | 57.9±5.6 |
| Male Sex (no. %) | 33 (51.56) | 34 (55.74) | 33 (54.10) | 34 (47.22) |
| BW (kg) | 68.7±10.8 | 69.4±10.4 | 68.0±12.6 | 68.6±10.3 |
| BMI (kg m^-2^) | 25.4±2.78 | 25.1±2.76 | 25.0±3.37 | 25.1±2.69 |
| WC (cm) | 89.9 ± 8.4 | 90.4 ± 7.5 | 89.2 ± 9.2 | 89.5 ± 8.2 |
| SBP (mmHg) | 129.0±15.6 | 129.8±13.9 | 129.6±15.2 | 127.7 ±12.3 |
| DBP (mmHg) | 78.7±8.5 | 79.9±8.1 | 79.6±9.5 | 78.9±8.3 |
| HbA1C (%)‡ | 7.78±0.79 | 7.65±0.68 | 7.67±0.76 | 7.61±0.76 |
| HbA1C (mmol mol**^-1^**) **§** | 61.53±14.85 | 60.11±16.07 | 60.33±15.19 | 59.67±15.19 |
| FPG (mmol L^-1^) | 7.90±1.25 | 8.28±1.49 | 8.24±1.40 | 8.08±1.12 |
| 2hPPG (mmolL L^-1^) | 14.86±2.54 | 14.86±3.24 | 14.59±3.23 | 14.66±2.90 |
| Fins (μIUml L^-1^) | 11.68 (7.74-17.21) | 10.12 (8.55-15.13) | 9.59 (6.70-14.49) | 10.16 (7.37-14.36) |
| ins120(μIUml L^-1^) | 55.11 (33.50-81.30) | 49.98 (34.33-64.60) | 53.93 (29.35-68.68) | 44.34 (31.27-70.58) |
| Fcp (ngml L^-1^) | 2.72 (2.01-3.39) | 2.50 (2.08-3.14) | 2.48 (1.92-2.95) | 2.39 (2.11-3.28) |
| cp120 (ng ml^-1^) | 8.17 (5.76-9.64) | 7.22 (6.03-9.08) | 7.38 (5.83-9.16) | 7.16 (5.67-9.85) |
| TG (mmol L^-1^) | 1.29 (0.97-1.75) | 1.46 (1.02-2.34) | 1.36 (1.03-2.07) | 1.59 (1.12-2.10) |
| TC(mmol L^-1^) | 5.15±1.02 | 5.36±1.09 | 5.01±1.04 | 5.31±0.95 |
| HDL(mmol L^-1^) | 1.33±0.28 | 1.24±0.28 | 1.30±0.30 | 1.22±0.24 |
| LDL (mmol L^-1^) | 3.29±0.87 | 3.54±0.89) | 3.22±0.89 | 3.47±0.81 |
| HOMA-IR (μIU·mol L^-2^) | 3.99 (2.68-5.74) | 4.04 (2.79-5.55) | 3.55 (2.38-4.97) | 3.51 (2.55-4.85) |
| HOMA-ß (IU·mol^-1^) | 55.37 (32.95-88.59) | 43.74 (33.55-70.59) | 42.23 (29.78-70.48) | 50.72 (31.62-68.23) |

BBR, berberine treatment; Prob, Probiotics treatment; Plac, Placebo; Prob+BBR: berberine plus probiotics treatment; BW: Body weight; BMI: Body mass index is the weight in kilograms divided by the square of the height in meter; WC: Waist circumference; SBP: Systolic blood pressure; DBP: Diastolic blood pressure; ‡HbA1c is glycated haemoglobin, shown as the DCCT (Diabetes Control and Complications Trial) units; **§**HbA1c is glycated haemoglobin, shown as the IFCC (International Federation of Clinical Chemistry) units; FPG: Fasting plasma glucose; 2hPPG: Post load plasma glucose; Fins: Fasting serum insuli; ins120: Post load serum insulin; Fcp: Fasting serum C peptide; cp120: Postload serum C peptide; TG: Triglyceride; TC: Total cholesterol; HDL: HDL cholesterol; LDL: LDL cholesterol; HOMA-IR: (Fasting serum insulin * Fasting plasma glucose)/22.5, homeostasis model assessment index for assessing insulin resistance; HOMA- ß: (20 * Fasting serum insulin)/(Fasting plasma glucose - 3.5), homeostasis model assessment index for assessing ß cell function; IQR, interquartile range; Mean±SD or Median (IQR).

Supplementary Table 8. Clinical Characteristics post Treatment in Subgroup Analysis (Age ≥50yrs)

|  | **Plac(n=60)** | | **Prob(n=60)** | | | **BBR(n=54)** | | | **Prob+BBR(n=70)** | | |
| --- | --- | --- | --- | --- | --- | --- | --- | --- | --- | --- | --- |
|  | **13w** | **Change¶** | **13w** | **Change¶** | **P** | **13w** | **Change¶** | **P** | **13w** | **Change¶** | **P** |
| BW (kg) | 67.5±10.7 | -1.73(-2.27,-1.19) | 67.9±10.0 | -1.63(-2.17,-1.09) | 0.84 | 66.7±12.2 | -1.80(-2.36,-1.23) | 0.95 | 66.6±10.5 | -1.76(-2.26,-1.26) | 0.79 |
| BMI (kg m^-2^) | 24.9±2.84 | -0.65(-0.85,-0.45) | 24.5±2.63 | -0.59(-0.79,-0.39) | 0.87 | 24.4±3.32 | -0.68(-0.89,-0.47) | 0.83 | 24.4±2.68 | -0.66(-0.85,-0.48) | 0.68 |
| WC (cm) | 88.6±8.3 | -1.51(-2.41,-0.61) | 88.7±7.20 | -1.73(-2.64,-0.83) | 0.71 | 86.9±8.8 | -2.37(-3.31,-1.42) | 0.14 | 87.4±7.5 | -1.91(-2.74,-1.07) | 0.32 |
| SBP (mmHg) | 125.5±12.7 | -3.92(-7.17,-0.67) | 124.9±12.6 | -5.10(-8.35,-1.85) | 0.66 | 128.7±13.4 | -2.62(-6.02,0.77) | 0.29 | 126.3±10.4 | -1.19(-4.19,1.82) | 0.23 |
| DBP (mmHg) | 75.1±8.5 | -3.50(-5.73,-1.27) | 76.1±8.2 | -3.88(-6.12,-1.65) | 0.87 | 78.6±9.9 | -1.71(-4.04,0.62) | 0.08 | 76.2±8.2 | -2.66(-4.72,-0.59) | 0.46 |
| FPG (mmol L^-1^) | 7.47±1.84 | -0.46(-0.77,-0.15) | 7.75±1.14 | -0.53(-0.83,0.22) | 0.75 | 6.88±0.96 | -1.38(-1.71,-1.06) | 1.48E-03 | 6.64±1.09 | -1.41(-1.70,-1.13) | 1.87E-05 |
| 2hPPG (mmolL L^-1^) | 14.08±3.78 | -0.70(-1.53,0.12) | 13.92±2.75 | -0.93(-1.76,0.10) | 0.66 | 11.45±2.66 | -3.25(-4.11,-2.39) | 7.01E-07 | 11.25±2.34 | -3.40(-4.16,-2.64) | 1.31E-08 |
| Fins (μIUml L^-1^) | 10.56(6.72-16.40) | -2.71(-4.58,-0.84) | 10.44(7.94-14.88) | -0.05(-1.90,1.81) | 0.12 | 9.47(5.52-12.72) | -1.54(-3.53,0.45) | 0.64 | 8.16(6.44-12.10) | -2.60(-4.32,-0.88) | 0.25 |
| ins120 (μIUml L^-1^) | 55.15(35.18-87.25) | 2.3(-3.6,8.2) | 51.13(35.10-70.56) | 4.46(-1.44,10.37) | 0.61 | 47.77(30.13-79.61) | 3.83(-2.39,10.05) | 0.83 | 46.23(34.07-78.76) | 3.48(-1.99,8.94) | 0.72 |
| Fcp(ngml L^-1^) | 2.39(2.00-3.27) | -0.36(-0.58,-0.15) | 2.45(1.95-3.14) | -0.12(-0.33,0.09) | 0.27 | 2.52(1.78-2.83) | -0.15(-0.38,0.07) | 0.95 | 2.16(1.84-2.92) | -0.27(-0.46,-0.07) | 0.62 |
| cp120 (ng ml^-1^) | 8.07(6.45-9.67) | 0.21(-0.24,0.66) | 7.19(5.90-8.93) | 0.31(-0.14,0.76) | 0.82 | 7.91(5.93-9.76) | 0.51(0.04,0.99) | 0.56 | 7.27(5.99-10.15) | 0.48(0.07,0.90) | 0.56 |
| TG (mmol L^-1^) | 1.34(1.04-1.72) | -0.03(-0.32,0.27) | 1.73(1.03-2.81) | 0.35(0.05,0.65) | 0.03 | 1.17(0.78-1.65) | -0.37(-0.68,-0.05) | 1.24E-03 | 1.27(1.01-1.91) | -0.32(-0.60,-0.05) | 0.04 |
| TC(mmol L^-1^) | 4.99±0.98 | -0.15(-0.36,0.06) | 5.21±1.12 | -0.17(-0.38,0.04) | 0.69 | 4.50±1.00) | -0.51(-0.73,-0.29) | 1.93E-03 | 4.70±0.98 | -0.61(-0.80,0.42) | 5.40E-04 |
| HDL(mmol L^-1^) | 1.31±0.31 | 0.01(-0.04,0.05) | 1.23±0.30 | -0.01(-0.06,0.03) | 0.31 | 1.28±0.26 | -0.02(-0.07,0.03) | 0.27 | 1.19±0.23 | -0.03(-0.07,0.01) | 0.08 |
| LDL (mmol L^-1^) | 3.21±0.95 | -0.09(-0.27,0.08) | 3.20±0.84 | -0.36(-0.53,-0.18) | 0.11 | 2.81±0.79 | -0.43(-0.61,-0.24) | 3.10E-03 | 2.95±0.84 | -0.53(-0.69,-0.37) | 1.05E-03 |
| HOMA-IR (μIU·mol L^-2^) | 3.46(2.07-5.1) | -1.39(-2.16,-0.61) | 3.68(2.46-5.30) | -0.28(-1.04,0.48) | 0.13 | 2.75(1.56-3.95) | -1.18(-2.00,-0.36) | 0.10 | 2.44(1.86-3.55) | -1.49(-2.20,-0.79) | 6.62E-03 |
| HOMA-ß (IU·mol^-1^) | 53.31(30.04-87.92) | 3.32(-7.04,13.67) | 49.41(38.59-70.73) | 3.28(-6.90,13.46) | 0.63 | 57.83(34.91-82.78) | 10.93(-0.01,21.87) | 0.11 | 58.79(37.88-82.21) | 10.49(1.06,19.92) | 0.05 |

BBR, berberine treatment; Prob, Probiotics treatment; Plac, Placebo, Prob+BBR: berberine plus probiotics treatment. BW: Body weight; BMI: Body mass index is the weight in kilograms divided by the square of the height in meter; WC: Waist circumference; SBP: Systolic blood pressure; DBP: Diastolic blood pressure; FPG: Fasting plasma glucose; PPG: Post load plasma glucose; Fins: Fasting serum insuli; 2hPPG: Post load plasma glucose; Fins: Fasting serum insulin; ins120: Post load serum insulin; Fcp: Fasting serum C peptide; cp120: Postload serum C peptide; TG: Triglyceride; TC: Total cholesterol; HDL: HDL cholesterol; LDL: LDL cholesterol; HOMA-IR: (Fasting serum insulin * Fasting plasma glucose)/22.5, homeostasis model assessment index for assessing insulin resistance; HOMA- ß: (20 * Fasting serum insulin)/(Fasting plasma glucose - 3.5), homeostasis model assessment index for assessing ß cell function. ¶The values are least-squares means represent changes from baseline (95%CI). Mean±SD or Median (IQR).

P value refers to comparison between each treatment group and placebo group using GEE model after treatment adjusted for baseline values and age (two-sided wald test).

Supplementary Table 9. Formula of the multi-strain probiotics

| Species | Strain | Biological Deposited No. |
| --- | --- | --- |
| *Bifidobacterium longum* | BL88-Onlly | CGMCC No.2107 |
| *Bifidobacterium breve* | BB8 | CGMCC No.6402 |
| *Lactococcus gasseri* | LG23 | CGMCC No.10758 |
| *Lactobacillus rhamnosus* | LR22 | CNCM I-4474 |
| *Lactobacillus salivarius* | LS86 | CGMCC No.6403 |
| *Lactobacillus crispatus* | LCR15 | CGMCC No.6406 |
| *Lactobacillus plantarum* | LP-Onlly | CGMCC No.1258 |
| *Lactobacillus fermentum* | LF33 | CGMCC No.6407 |
| *Lactobacillus casei* | LC18 | CNCM I-4458 |

CGMCC, China General Microbiological Culture Collection Centre

CNCM, Collection Nationale De Cultures De Microorganisms, France

Supplementary Table 10. Formula of culture medium for *R.bromii*

| **Medium. MPYG medium** | |
| --- | --- |
| **Component** | **Amount/L** |
| Trypticase peptone | 5.00 g |
| Peptone | 3.00 g |
| Peptone from soya | 2.00 g |
| Polypeptone | 1.00 g |
| Yeast extract | 10.00 g |
| Beef extract | 5.00 g |
| Glucose | 5.00 g |
| Tween 80 | 0.50 ml |
| Maltose | 0.50 g |
| Cellobiose | 0.50 g |
| Starch, soluble | 0.50 g |
| Glycerol | 0.50 ml |
| K2HPO4 | 2.00 g |
| Cysteine-HCl x H2O | 0.50 g |
| Na2S | 0.25 g |
| Resazurin | 1.00 mg |
| Salt solution (see below) | 40.00 ml |
| Trace element(see below) | 10.00 ml |
| Vitamin solution(see below) | 10.00 ml |
| Haemin solution (see below) | 10.00 ml |
| Vitamin K1 solution (see below) | 0.20 ml |
| Agar | 15 g |
|  |  |
| **Salt solution(DSMZ Salt solution):** |  |
| CaCl2 x 2 H2O | 0.25 g |
| MgSO4 x 7 H2O | 0.50 g |
| K2HPO4 | 1.00 g |
| KH2PO4 | 1.00 g |
| NaHCO3 | 10.00 g |
| NaCl | 2.00 g |
| Distilled water | 1000.00 ml |
| **Trace element solution (DSMZ Trace element solution):** | |
| Nitrilotriacetic acid | 1.50 g |
| MgSO4 x 7 H2O | 3.00 g |
| MnSO4 x H2O | 0.50 g |
| NaCl | 1.00 g |
| FeSO4 x 7 H2O | 0.10 g |
| CoSO4 x 7 H2O | 0.18 g |
| CaCl2 x 2 H2O | 0.10 g |
| ZnSO4 x 7 H2O | 0.18 g |
| CuSO4 x 5 H2O | 0.01 g |
| KAl(SO4)2 x 12 H2O | 0.02 g |
| H3BO3 | 0.01 g |
| Na2MoO4 x 2 H2O | 0.01 g |
| NiCl2 x 6 H2O | 0.03 g |
| Na2SeO3 x 5 H2O | 0.30 mg |
| Distilled water | 1000.00 ml |
|  |  |
| **Vitamin solution:** |  |
| Biotin | 2.00 mg |
| Folic acid | 2.00 mg |
| Pyridoxine-HCl | 10.00 mg |
| Thiamine-HCl x 2 H2O | 5.00 mg |
| Riboflavin | 5.00 mg |
| Nicotinic acid | 5.00 mg |
| D-Ca-pantothenate | 5.00 mg |
| Vitamin B12 | 0.10 mg |
| p-Aminobenzoic acid | 5.00 mg |
| Lipoic acid | 5.00 mg |
| Distilled water | 1000.00 ml |


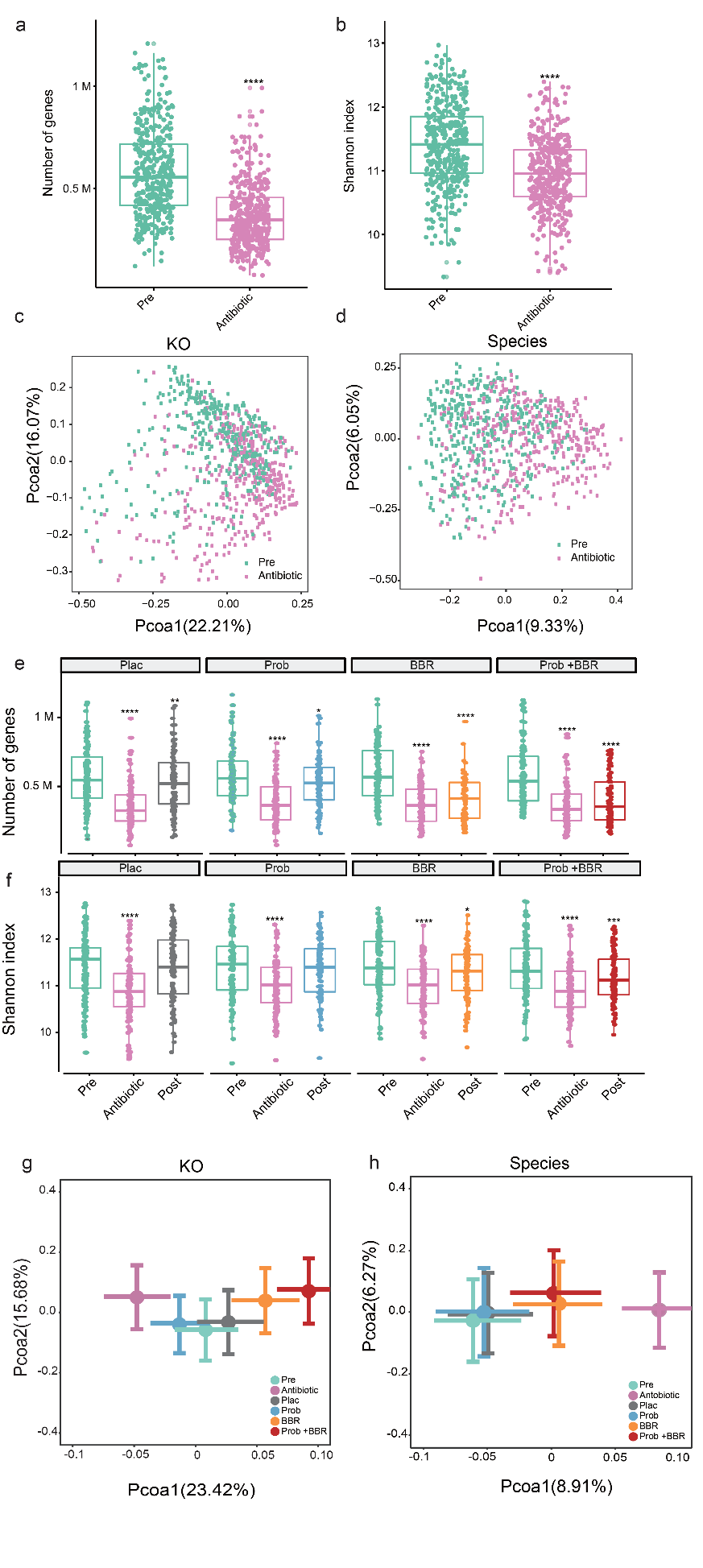
Supplementary Figure 1. **Comparison of the gut microbiota alterations after gentamycin and antidiabetic treatment.** Bar plots showed that gut microbial gene numbers (**a**) and gene-level Shannon index (**b**) were significantly decreased after single antibotics, gentamycin treatment in all participants. Baseline (green,), after 1-week gentamycin treatment (pink), Wilcoxon signed rank Test. Principal coordinate analysis (PCoA) based on Bray-Curtis dissimlarities at the species **(c)** and KOs (KEGG Orthology) (**d**) in all participants at baseline (green) and after 1-week gentamycin treatment (pink). Bar plots of gut microbial gene numbers (**e**) and gene-level Shannon index (**f**) in participants of 4 treatment arms in different time points (Plac, gray, n=96), probiotics (Prob, blue, n=98), berberine (BBR, orange, n=85) and probiotics plus berberine (Prob+BBR, scarlet, n=102). Two-sided wilcoxon signed rank Test, ****, P<0.0001, ***,P<0.001, **,P<0.01,*, P<0.05, versus baseline. Dark lines in the boxes indicate medians, the width of the notches is the IQR, the lowest and highest values within 1.5 times the IQR from the first and third quartiles. PCoA at the species (**g**) and KO (**h**) level of participants at baseline after 1-week gentamycin treatment and after 3 months treatments of Plac, Prob, BBR and Prob+BBR. Baseline, samples from all participants collected at baseline; Antibiotic, samples from all participants collected after gentamycin treatment; Plac, samples collected after 3 months treatment with placebo, gray; Prob, samples collected after 3 months treatment with probiotics, blue; BBR, samples collected after treatment with BBR, orange; Prob+BBR, samples collected after treatment with Probiotics plus BBR arm, scalet. Circles indicate the the mean coordinates of the participants in the group. Data are shown as means ± SD. Underlying data and exact P value are provided in the Source Data file.
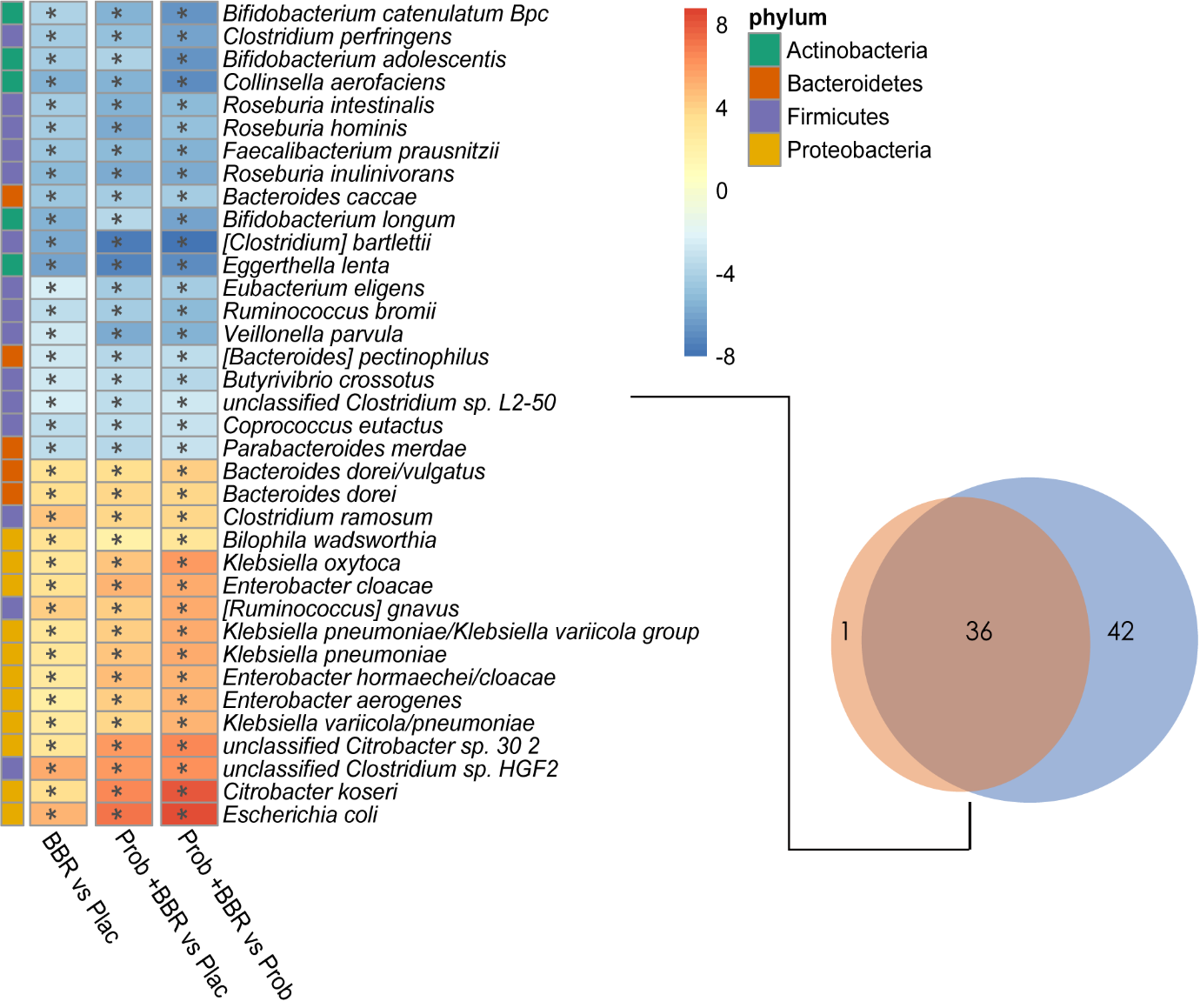
Supplementary Figure 2. **Key BBR responding Species.** Left, Heatmap of post treatment RA of key BBR responding species which showed signficant differences in all comparisons (BBR vs Plac, BBR+Prob vs Prob and BBR+Prob vs Plac), and significantly changed their RA after BBR or BBR+Prob treatment. Color key: Z score, *q<0.05, Z score <0, indicated decreased compared to Plac or Prob , Z score >0 indicated increased compared to plac or Prob , Kruskal-wallis test. Right, Venn map: Blue circle indicates number of species that showed significant alterations in the relative abundance at baseline and after treatment in BBR or BBR+Prob, Two-sided wilcoxon signed rank test, q < 0.05, n= 78 taxa. Orange circle indicates number of species that showed consistent and significant differences in the post-treatment relative abundance in three comparisions inclduing BBR vs Placebo, BBR+Prob vs Prob, and BBR+Prob vs Plac, n=37 taxa. Kruskal-Wallis test, Dunn’s P<0.05, two-sided. *Bifidobacterium catenulatum−Bpc: Bifidobacterium catenulatum-Bifidobacterium pseudocatenulatum complex*. Underlying data and exact P value are provided in the Source Data file.
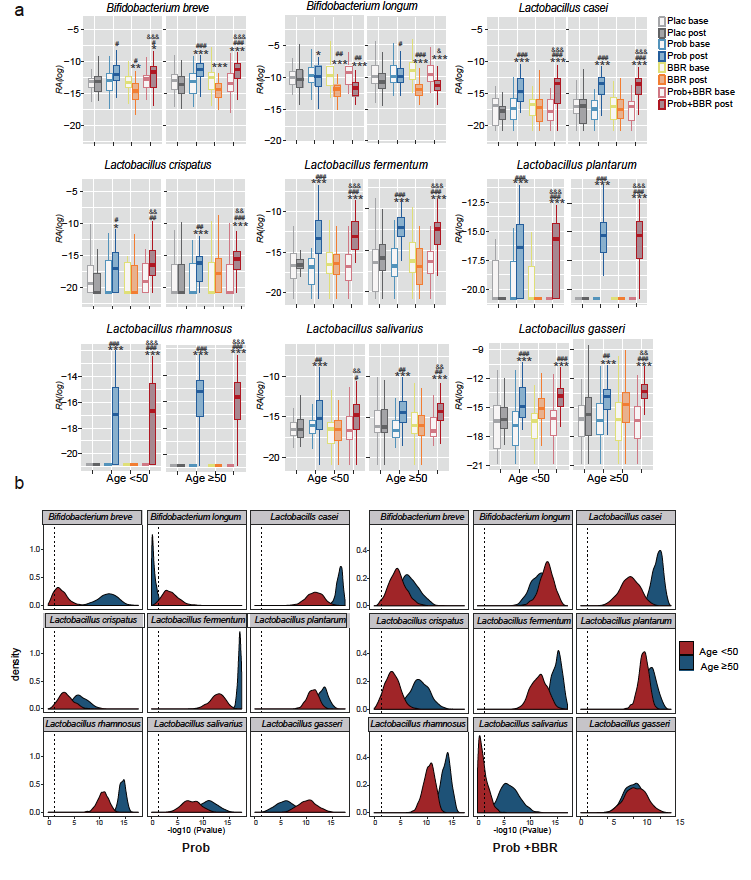
Supplementary Figure 3. **Probiotics enrichment after intervention was different in two age subgroups. (a)** Bar-plot showed the relative abundances (RAs) of the 9 probiotics in participants (age ≥50 or <50) at baseline and post treatment. *, P<0.05, **, P<0.01, ***, P<0.001, baseline vs post-treatment, Wilcoxon signed rank test, two-sided. #, P<0.05, ##, P<0.01, ###, P<0.001 treatment arms vs the Plac, &, P<0.05, &&, P<0.01, &&&, P<0.001 treatment arms vs the BBR, Kruskal-wallis test and *Dunn’s post hoc* test, two-sided. Dark lines in the boxes indicate medians, the width of the notches is the IQR, the lowest and highest values within 1.5 times the IQR from the first and third quartiles. Age <50：Plac, n= 37; Prob, n=39; BBR, n=34; Prob+BBR: n=33. Age ≥50: Plac, n=59; Prob, n= 59; BBR, n=51; Prob+BBR: n=69. **(b)** Density curves showed the distribution of the P value in two age groups (red, < 50 yrs; blue ≥50 yrs) in Prob and Prob+BBR treatment arms. P values were calcuated based on comparisons of relative abundance of 9 probiotic species between baseline and post-treatment in each group (Wilcoxon signed rank test, two-sided). To evaluate whether the different sample sizes between the age subgroups would bring bias to comparison, 100 bootstrap paired samples were generated by randomly resampling with replacement for each age group. A total of 1000 replications were simulated for each comparison, and most of which showed a smaller P value in older subgroup. base: baseline, post: post treatment. Underlying data and exact P value are provided in the Source Data file.
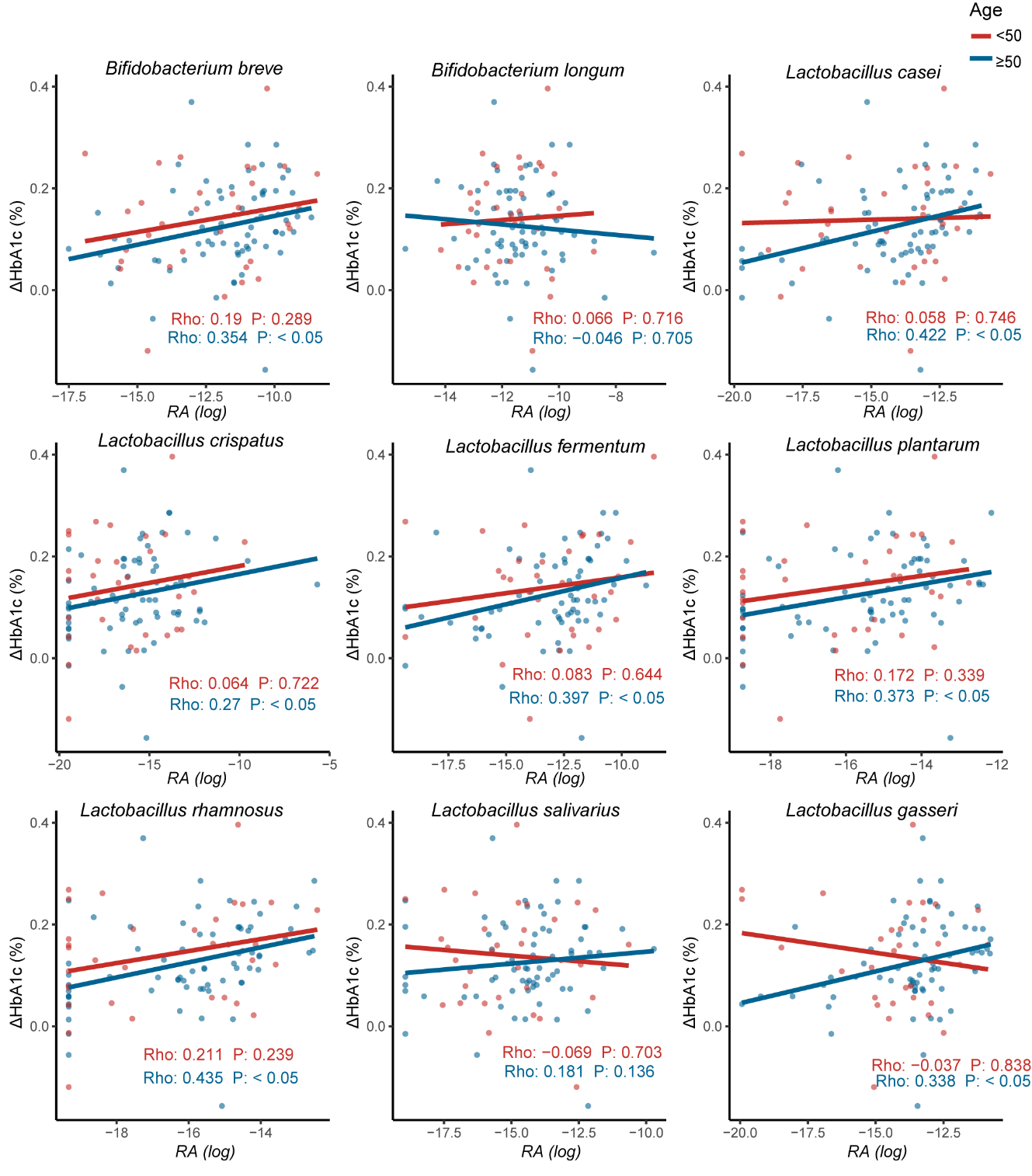
Supplementary Figure 4. **Correlation of HbA1c reduction with Post-treatment abundance of 9 probiotic species.** In samples from participants (age ≥50 or <50) treated with Prob +BBR, the correlations between the Δ HbA1c and post treatment relative abundances (RAs) of 9 probiotic containing species were plotted in two age subgroups. Δ HbA1c=100%*(baseline value of HbA1c- posttreatment value of HbA1c)/ baseline value of HbA1c; X axis represented the log-transformed post-treatment RAl of each species; Rho represented Spearman correlation coefficient. Red, age of participants<50, n=33; Blue, age of participants≥50, n=69. Underlying data and exact P value are provided in the Source Data file.


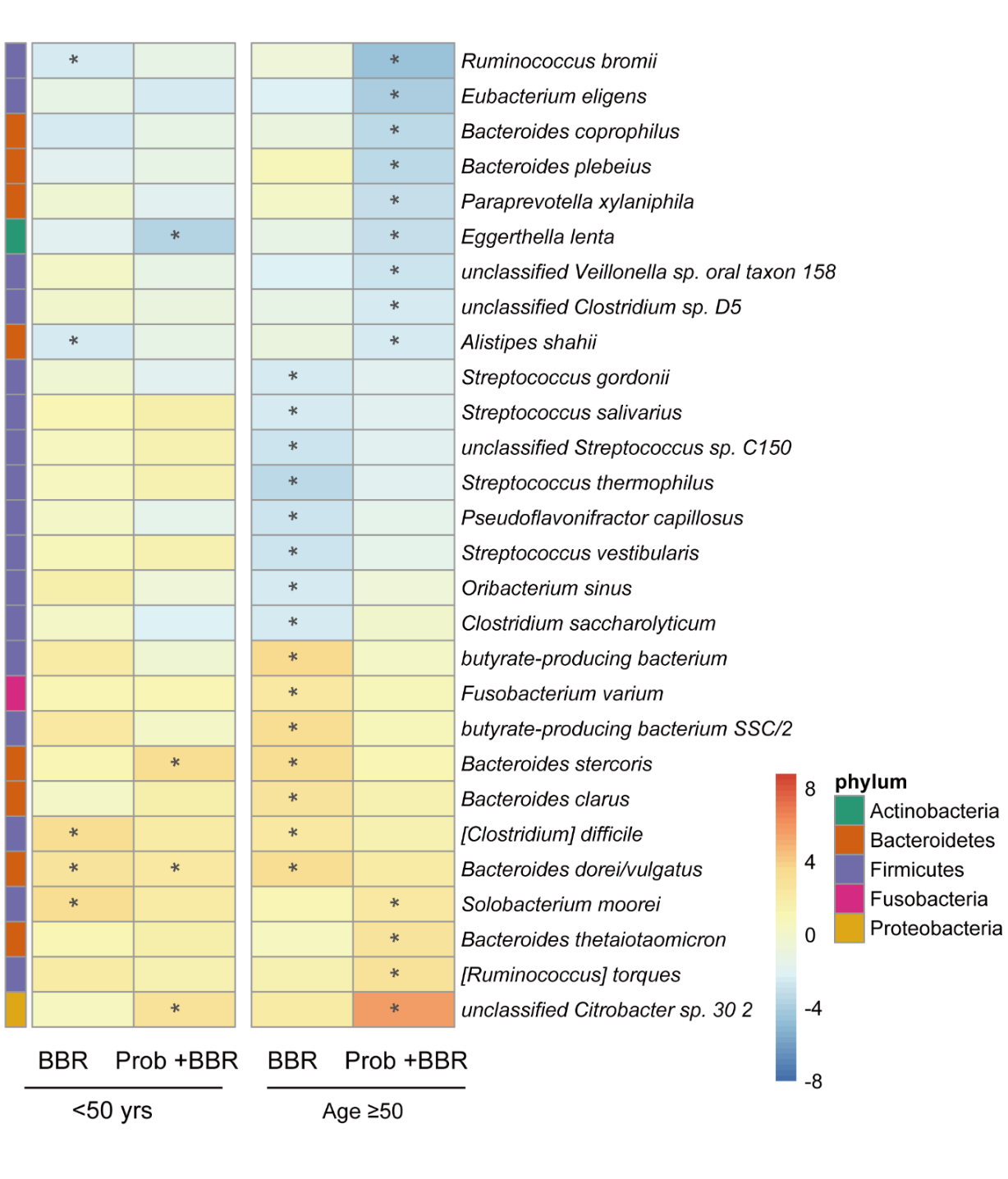


## Supplementary Figure 5. Heatmap of gut microbiota that differentially altered from baseline in older participants treated by BBR and Prob+BBR. The heatmap showed the changes of species relative abundance (RA) between baseline and post treatment in BBR and Prob+BBR arms of different age groups, ≥50 or <50. Only species showed different alterations between BBR and Prob+BBR were plotted. Color key, Z score was calculated with Wilcoxon signed rank tests, Z score <0, enriched at the baseline; Z score >0 enriched post treatment. *, q<0.05, two-sided wilcoxon signed rank test. Age <50:BBR, n=34; Prob+BBR: n=33. Age ≥50: BBR, n=51; Prob+BBR: n=69. Underlying data and exact P value are provided in the Source Data file.**
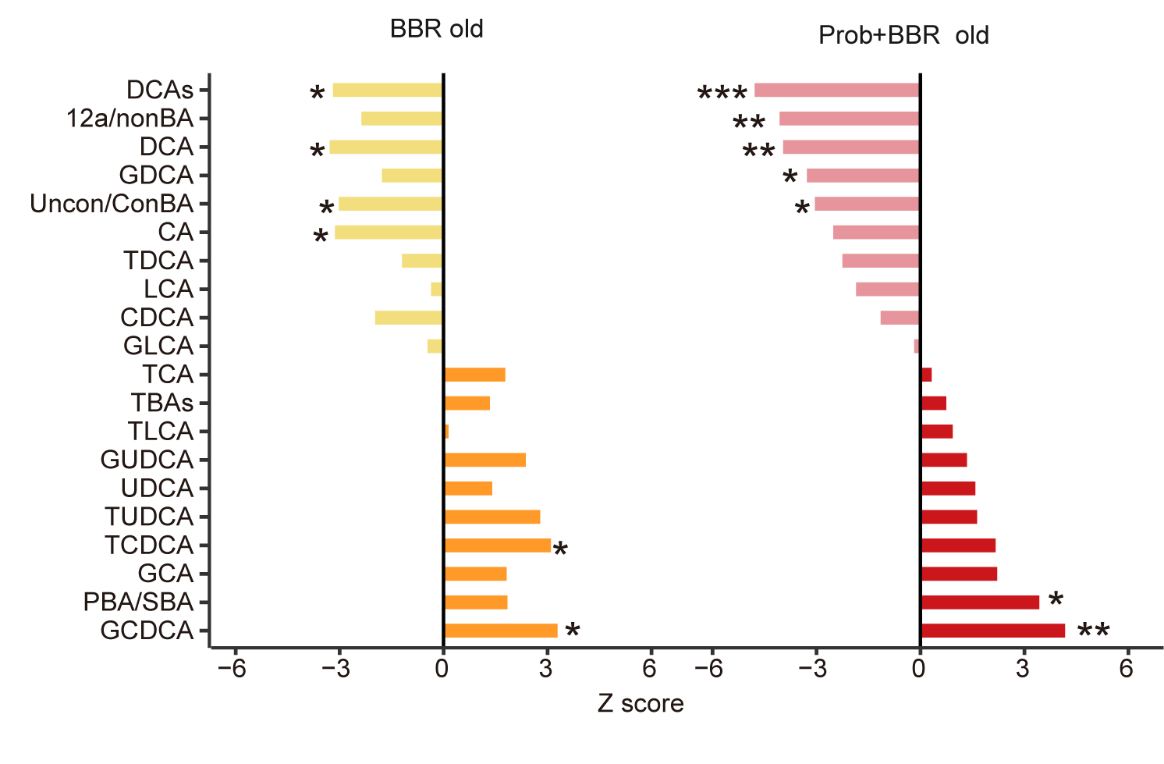
Supplementary Figure 6.** BA alterations between two BBR arms in older participant. Bar plot showing the changes in blood bile acid (BA) percentage between baseline and post treatment in BBR and Prob+BBR arms of participants ≥50 years. Z score was calculated with two-sided wilcoxon signed rank tests; Z score < 0, enriched at baseline ; Z score > 0 enriched post treatment. *, q<0.01, **, q<0.01, ***, q<0.001. DCAs, Total deoxycholic acid; 12a/nonBA, 12a-hydroxylated/non–12a-hydroxylated bile acids; DCA, deoxycholic acid; GDCA, glycodeoxycholic acid; Uncon/ConBA, unconjugated/conjugated bile acids; CA, cholic acid; TDCA, taurodeoxycholic acid; LCA, lithocholic acid; CDCA, chenodeoxycholic acid; GLCA, glycolithocholic acid; TCA, taurocholic acid; TBAs, Total bile acids; TLCA, taurolithocholic acid; GUDCA, glycoursodeoxycholic acid; UDCA, ursodeoxycholic acid; TUDCA, tauroursodeoxycholic acid; TCDCA, taurocholic chenodeoxycholic acid; GCA, glycocholic acid; PBA/SBA, Primary/Secondary bile acids; GCDCA, glycochenodeoxycholic acid. BBR old: n=51; Prob+BBR old: n=69. Underlying data and exact P value are provided in the Source Data file.

# **Supplementary Note 1**

# CONSORT 2010 checklist of information to include when reporting a randomised trial*

| Section/Topic | Item No | Checklist item | 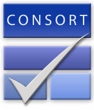Reported on page No |
| --- | --- | --- | --- |
| Title and abstract | | | |
|  | 1a | Identification as a randomised trial in the title | N/A |
|  | 1b | Structured summary of trial design, methods, results, and conclusions (for specific guidance see CONSORT for abstracts) | 2 |
| Introduction | | | |
| Background and objectives | 2a | Scientific background and explanation of rationale | 3 |
|  | 2b | Specific objectives or hypotheses | 4 |
| Methods | | | |
| Trial design | 3a | Description of trial design (such as parallel, factorial) including allocation ratio | 10-11,18, Figure1 |
|  | 3b | Important changes to methods after trial commencement (such as eligibility criteria), with reasons | N/A |
| Participants | 4a | Eligibility criteria for participants | 10-11 |
|  | 4b | Settings and locations where the data were collected | 10, 13-14 |
| Interventions | 5 | The interventions for each group with sufficient details to allow replication, including how and when they were actually administered | 10-11 |
| Outcomes | 6a | Completely defined pre-specified primary and secondary outcome measures, including how and when they were assessed | 14-15 |
|  | 6b | Any changes to trial outcomes after the trial commenced, with reasons | N/A |
| Sample size | 7a | How sample size was determined | 18 |
|  | 7b | When applicable, explanation of any interim analyses and stopping guidelines | 11-13,18, Figure1 |
| Randomisation: |  |  |  |
| Sequence generation | 8a | Method used to generate the random allocation sequence | 10-11 |
|  | 8b | Type of randomisation; details of any restriction (such as blocking and block size) | 10-11 |
| Allocation concealment mechanism | 9 | Mechanism used to implement the random allocation sequence (such as sequentially numbered containers), describing any steps taken to conceal the sequence until interventions were assigned | 10-11 |
| Implementation | 10 | Who generated the random allocation sequence, who enrolled participants, and who assigned participants to interventions | 10-11, 23 |
| Blinding | 11a | If done, who was blinded after assignment to interventions (for example, participants, care providers, those assessing outcomes) and how | 11 |
|  | 11b | If relevant, description of the similarity of interventions | 4, 11 |
| Statistical methods | 12a | Statistical methods used to compare groups for primary and secondary outcomes | 18 |
|  | 12b | Methods for additional analyses, such as subgroup analyses and adjusted analyses | 18 |
| Results | | | |
| Participant flow (a diagram is strongly recommended) | 13a | For each group, the numbers of participants who were randomly assigned, received intended treatment, and were analysed for the primary outcome | 4, Figure 1 |
|  | 13b | For each group, losses and exclusions after randomisation, together with reasons | Figure 1 |
| Recruitment | 14a | Dates defining the periods of recruitment and follow-up | 4,10 |
|  | 14b | Why the trial ended or was stopped | N/A |
| Baseline data | 15 | A table showing baseline demographic and clinical characteristics for each group | 24-25 |
| Numbers analysed | 16 | For each group, number of participants (denominator) included in each analysis and whether the analysis was by original assigned groups | Figure 1, 24-27 |
| Outcomes and estimation | 17a | For each primary and secondary outcome, results for each group, and the estimated effect size and its precision (such as 95% confidence interval) | 18, 24-26 Supplementary Information |
|  | 17b | For binary outcomes, presentation of both absolute and relative effect sizes is recommended | N/A |
| Ancillary analyses | 18 | Results of any other analyses performed, including subgroup analyses and adjusted analyses, distinguishing pre-specified from exploratory | 4-5, 26, Supplementary Information |
| Harms | 19 | All important harms or unintended effects in each group (for specific guidance see CONSORT for harms) | 2, 4-5, Supplementary information |
| Discussion | | | |
| Limitations | 20 | Trial limitations, addressing sources of potential bias, imprecision, and, if relevant, multiplicity of analyses | 10 |
| Generalisability | 21 | Generalisability (external validity, applicability) of the trial findings | 9 |
| Interpretation | 22 | Interpretation consistent with results, balancing benefits and harms, and considering other relevant evidence | 2, 8-10 |
| Other information | | |  |
| Registration | 23 | Registration number and name of trial registry | 2,4 |
| Protocol | 24 | Where the full trial protocol can be accessed, if available | Uploaded in Nature communications manuscript system |
| Funding | 25 | Sources of funding and other support (such as supply of drugs), role of funders | 23 |

*We strongly recommend reading this statement in conjunction with the CONSORT 2010 Explanation and Elaboration for important clarifications on all the items. If relevant, we also recommend reading CONSORT extensions for cluster randomised trials, non-inferiority and equivalence trials, non-pharmacological treatments, herbal interventions, and pragmatic trials. Additional extensions are forthcoming: for those and for up to date references relevant to this checklist, see [www.consort-statement.org](http://www.consort-statement.org).
