# Supplementary material for: Gut microbiome-related effects of berberine and probiotics on type 2 diabetes (the PREMOTE study)
Source: Nat Commun. 2020 Oct 6;11:5015. doi: 10.1038/s41467-020-18414-8 (PMC7538905; doi:10.1038/s41467-020-18414-8)
Supplement: Supplementary file 2 — Description of Additional Supplementary Files [file 41467_2020_18414_MOESM2_ESM.docx]

**Description of Additional Supplementary Files**

**Title: Supplementary Data file 1**

**Description:** Profile of species altered in different time points and treatment arms (Spreadsheet) Wilcoxon matched-pairs signed rank tests (two-sided test) were applied to detect differences between baseline versus after 1-week gentamycin treatment (Antibiotic) in all participants, and baseline versus post treatment in each treatment arm (Plac, placebo treatment arm; Prob, probiotics treatment arm; BBR, BBR treatment arm; Prob+BBR, Probiotics plus BBR treatment arm). q value < 0.05 was considered significant.

**Title: Supplementary Data file 2**

**Description:** Profile of probiotics containing species altered between baseline and post treatment in all arms (Spreadsheet) Wilcoxon matched-pairs signed rank tests (two-sided test) were applied to detect differences between baseline versus post treatment in each treatment arm (Plac, placebo treatment arm; Prob, probiotics treatment arm; BBR, BBR treatment arm; Prob+BBR, Probiotics plus BBR treatment arm). q value < 0.05 was considered significant.

**Title: Supplementary Data file 3**

**Description:** Comparison of posttreatment RAs of key BBR responding species between 4 arms (Spreadsheet) Kruskal–Wallis tests were applied to detect the differences between 4 groups. Dunn’s post hoc tests (two-sided test) were further performed to explore the differences between two groups. Dunn’s P value less than 0.05 was considered significant.

**Title: Supplementary Data file 4**

**Description:** KEGG pathway and module enrichment analysis (Spreadsheet) Differential enrichment of KEGG pathways/modules in post treatment individuals between groups were identified according to the reporter Z scores of all detected Kos involved in the given pathway (module). An absolute value of reporter score ≥ 1.96 was used as the detection threshold for significance. Plac, placebo treatment arm; Prob, probiotics treatment arm; BBR, BBR treatment arm; Prob+BBR, Probiotics plus BBR treatment arm.

**Title:** **Supplementary Data file 5.**

**Description:** Blood Bile acid profile analysis (Spreadsheet) Wilcoxon signed rank tests (two-sided test) were applied to detect differences of blood bile acid (BA) percentage and total BA amount (TBAs) between baseline and post treatment in each treatment arm. q<0.05 was considered significant. CA, cholic acid; CDCA, chenodeoxycholic acid; DCA, deoxycholic acid; GCA, glycocholic acid; GCDCA, glycochenodeoxycholic acid; GDCA, glycodeoxycholic acid; GLCA, glycolithocholic acid; GUDCA, glycoursodeoxycholic acid; LCA, lithocholic acid; TCA, taurocholic acid; TCDCA, taurocholic chenodeoxycholic acid; TDCA, taurodeoxycholic acid; TLCA, taurolithocholic acid; TUDCA, tauroursodeoxycholic acid; UDCA, ursodeoxycholic acid; Uncon/ConBA, unconjugated/conjugated bile acids; PBA/SBA, Primary/Secondary bile acids; 12a/nonBA, 12a-hydroxylated/non–12a-hydroxylated bile acids; DCAs, Total deoxycholic acid; TBAs, Total bile acids.

**Title:** **Supplementary Data file 6.**

**Description:** Correlation between plasma BAs and major clinical parameters in BBR or Prob+BBR treatment arms (Spreadsheet) Multivariate GEE analysis was performed to estimate the correlations between changes in percentage of the blood bile acids with change in major clinical outcomes controlling for age, sex and BMI, estimate represented β value. BBR, BBR treatment arm; Prob+BBR, Probiotics plus BBR treatment arm. HbA1c, glycated haemoglobin; FPG, Fasting plasma glucose; 2hPPG, Post load plasma glucose; ins120, Post load serum insulin; cp120, Post load serum C peptide; HOMA-IR, homeostasis model assessment index for assessing insulin resistance; HOMA- ß, homeostasis model assessment index for assessing ß cell function; TC, Total cholesterol; TG, triglycerides; LDLC, LDL cholesterol; CA, cholic acid; CDCA, chenodeoxycholic acid; DCA, deoxycholic acid; GCA, glycocholic acid; GCDCA, glycochenodeoxycholic acid; GDCA, glycodeoxycholic acid; GLCA, glycolithocholic acid; GUDCA, glycoursodeoxycholic acid; LCA, lithocholic acid; TCA, taurocholic acid; TCDCA, taurocholic chenodeoxycholic acid; TDCA, taurodeoxycholic acid; TLCA, taurolithocholic acid; TUDCA, tauroursodeoxycholic acid; UDCA, ursodeoxycholic acid; Uncon/ConBA, unconjugated/conjugated bile acids; PBA/SBA, Primary/Secondary bile acids; 12a/nonBA, 12a-hydroxylated/non–12a-hydroxylated bile acids; DCAs, Total deoxycholic acid; TBAs, Total bile acids.

**Title:** **Supplementary Data file 7**

**Description:** The raw data of metabolome study for determining serum bile acid pool components.
