# Supplementary material for: Gut microbiome-related effects of berberine and probiotics on type 2 diabetes (the PREMOTE study)
Source: Nat Commun. 2020 Oct 6;11:5015. doi: 10.1038/s41467-020-18414-8 (PMC7538905; doi:10.1038/s41467-020-18414-8)
Supplement: Supplementary file 10 — Reporting Summary [file 41467_2020_18414_MOESM10_ESM.pdf]

nature research

Corresponding author(s): NCOMMS-19-18639-T

Last updated by author(s): May 6, 2020

Reporting Summary

Nature Research wishes to improve the reproducibility of the work that we publish. This form provides structure for consistency and transparency in reporting. For further information on Nature Research policies, see Authors & Referees and the Editorial Policy Checklist .

Statistics

For all statistical analyses, confirm that the following items are present in the figure legend, table legend, main text, or Methods section.

n/a	Confirmed	
☐	☒	The exact sample size (*n*) for each experimental group/condition, given as a discrete number and unit of measurement
☐	☒	A statement on whether measurements were taken from distinct samples or whether the same sample was measured repeatedly
☐	☒	The statistical test(s) used AND whether they are one- or two-sided<br>*Only common tests should be described solely by name; describe more complex techniques in the Methods section.*
☐	☒	A description of all covariates tested
☐	☒	A description of any assumptions or corrections, such as tests of normality and adjustment for multiple comparisons
☐	☒	A full description of the statistical parameters including central tendency (e.g. means) or other basic estimates (e.g. regression coefficient) AND variation (e.g. standard deviation) or associated estimates of uncertainty (e.g. confidence intervals)
☐	☒	For null hypothesis testing, the test statistic (e.g. *F*, *t*, *r*) with confidence intervals, effect sizes, degrees of freedom and *P* value noted<br>*Give P values as exact values whenever suitable.*
☒	☐	For Bayesian analysis, information on the choice of priors and Markov chain Monte Carlo settings
☐	☒	For hierarchical and complex designs, identification of the appropriate level for tests and full reporting of outcomes
☐	☒	Estimates of effect sizes (e.g. Cohen's *d*, Pearson's *r*), indicating how they were calculated

Our web collection on statistics for biologists contains articles on many of the points above.

Software and code

Policy information about availability of computer code

Data collection	Metagenomics data were collected using IGC gene catalogs (http://meta.genomics.cn/meta/dataTools).
Data analysis	The following softwares were used: SOAP v2.22, BLAST v2.2.24, SAS v9.4 (SAS Institute, Cary, NC, USA), Skyline v4.2<br>These following R 3.3.2 packages were used: ape 5.3, vegan 2.5-6, geeM 0.10.1, pheatmap 1.0.12, coin 1.3-1, ggplot2 3.2.1, PMCMR 4.3.

For manuscripts utilizing custom algorithms or software that are central to the research but not yet described in published literature, software must be made available to editors/reviewers. We strongly encourage code deposition in a community repository (e.g. GitHub). See the Nature Research guidelines for submitting code & software for further information.

Data

Policy information about availability of data

All manuscripts must include a data availability statement. This statement should provide the following information, where applicable:

- Accession codes, unique identifiers, or web links for publicly available datasets
- A list of figures that have associated raw data
- A description of any restrictions on data availability

Metagenomic sequencing data for the 1192 faecal samples can be accessed from the China Nucleotide Sequence Archive (CNSA) with the dataset identifier CNP0000478 (https://db.cngb.org/search/?q=CNP0000478) and the National Center for Biotechnology Information BioProject Database with the dataset accession number PRJNA643353 (https://www.ncbi.nlm.nih.gov/bioproject/PRJNA643353). The metabolomics raw data was shown in Supplementary Data file 7. The other datasets analysed in this study were available at KEGG Release 87.0 (https://www.genome.jp/kegg-bin/) and at IGC (http://meta.genomics.cn/meta/dataTools). All other data are available upon request. The source data underlying Figs.2, 3, and 4, and Supplementary Figs.1, 2, 3, 4, 5, and 6 are provided as a source data file.

Field-specific reporting

Please select the one below that is the best fit for your research. If you are not sure, read the appropriate sections before making your selection.

- [x] Life sciences
- [ ] Behavioural & social sciences
- [ ] Ecological, evolutionary & environmental sciences

For a reference copy of the document with all sections, see nature.com/documents/nr-reporting-summary-flat.pdf

Life sciences study design

All studies must disclose on these points even when the disclosure is negative.

Sample size	The study sample size calculations were based on previously published data (Ref: Tonucci LB, et al. Clin Nutr. 2017;36:85-92, and Zhang Y, et al. J Clin Endocrinol Metab. 2008;93:2559-2565. and the primary outcome in the current study, which is change in HbA1c. The sample size estimation is within the framework of generalized estimating equations (GEE) models (Ref: Jung SH, et al. Stat Med. 2003;22:1305-1315). For primary outcome: The outcome variable is HbA1c with a baseline measurement and the follow-up at 13 weeks after intervention initiation. The aim of the study is a comparison of slopes in repeated measurements with equal allocation in 4 treatment arms. Based on the preliminary data, with a sample size of 360 study participants, this study will have a power of 86% (based on a two-sided test, α = 5%). We have conservatively assumed that the overall dropout rate will be 10% during the 13-week study period. To account for loss to follow-up, the power will be 86% if 400 study subjects are recruited. Detailed sample size calculation was summarized in Method and Study protocol.
Data exclusions	Statistical analyses for clinical data includes all subjects who are randomized independent of whether they received study treatment or not. Samples that were not paired pre and post treatment would not be included in the paired Wilcox study and multi-omics analysis.
Replication	For clinical study, a total of 566 patients were screened for eligibility from August 18, 2016 to July 18, 2017, of whom 409 participants underwent randomization: 106 were randomly assigned to the Prob+BBR group, 102 to Prob group, 98 to the BBR group, and 103 to the Plac group. For the in vitro studies, at least 3 replicates in 3 independent experiments were performed in each group.
Randomization	Participants were randomly assigned into the treatment groups. The randomization procedure was stratified by age, in a block size of 8 and generated utilizing a validated interactive Web-based Response System (IWRS) which was maintained by an independent data manager.
Blinding	The study personnel and participants were blinded to the assignment of treatment groups. The study investigators and statisticians were all blinded during the study procedure before the database lock, and were unblinded after database unblinding procedure.

Reporting for specific materials, systems and methods

We require information from authors about some types of materials, experimental systems and methods used in many studies. Here, indicate whether each material, system or method listed is relevant to your study. If you are not sure if a list item applies to your research, read the appropriate section before selecting a response.

Materials & experimental systems

n/a	Involved in the study	
x		Antibodies
x		Eukaryotic cell lines
x		Palaeontology
x		Animals and other organisms
	x	Human research participants
	x	Clinical data

Methods

n/a	Involved in the study	
x		ChIP-seq
x		Flow cytometry
x		MRI-based neuroimaging

Human research participants

Policy information about studies involving human research participants

Population characteristics	The participants were those with newly diagnosed T2DM according to the World Health Organization criteria, drug naïve for glycaemic control but with at least 2 months of stable life-style intervention; aged between 20 and 70 years, with both genders eligible; had a body-mass index (BMI) from 19.0 to 35.0 kg/m², with glycated haemoglobin (HbA1c) ≥ 6.5% and ≤ 10.0%, and fasting plasma glucose (FPG) ≥ 7.0mmol/L and ≤ 13.3mmol/L at screening. Detailed inclution criteria are seen in Study protocol.
Recruitment	Between August 18, 2016 and July 18, 2017, consecutive T2D patients from the outpatient department at each study site, who were willing to participant in the current study and signed informed consent form, were screened for eligibility and totally 409 participants were enrolled. Therefore, no systematic selection bias was present in the current study. Detailed inclusion and exclusion criteria were summarized in Supplementary material and Study protocol.
Ethics oversight	The trial conformed to the provisions of the Declaration of Helsinki and was approved by the ethics committees at Ruijin Hospital, Shanghai Jiao Tong University School of Medicine, the leading center of the present study, and at the other 19 participating centres subsequently. All the participants provided written informed consent.

Note that full information on the approval of the study protocol must also be provided in the manuscript.

Clinical data

Policy information about clinical studies

All manuscripts should comply with the ICMJEguidelines for publication of clinical research and a completedCONSORT checklist must be included with all submissions.

Clinical trial registration	ClinicalTrials.gov number, NCT02861261
Study protocol	Study protocol can be accessed in the Supplementary material
Data collection	Data were recorded on paper CRF and sent to the data center and entry into the database system. Laboratory and metagenomic/metabonomic data were sent to the sponsor (study leading center) directly from the vendors/collaborators. All data were source verified, reviewed, queried and cleaned before database lock. Quality of study data was assured through monitoring of study sites, provisions of appropriate trainings for study personnel, and use of data management procedures. The study was carried out in accordance with GCP guidelines.
Outcomes	The clinical outcomes included the improvement of glycaemic control, defined as the changes in HbA1c levels, as the primary outcome, and the changes in fasting or post load blood glucose, lipids, insulin, homeostasis model assessment index for insulin resistance (HOMA-IR), and ß cell function (HOMA-ß), as the secondary outcomes during the follow-up.
